# RNF8/UBC13 ubiquitin signaling suppresses synapse formation in the mammalian brain

**DOI:** 10.1038/s41467-017-01333-6

**Published:** 2017-11-02

**Authors:** Pamela Valnegri, Ju Huang, Tomoko Yamada, Yue Yang, Luis A. Mejia, Ha Y. Cho, Anna Oldenborg, Azad Bonni

**Affiliations:** 1 Department of Neuroscience, Washington University School of Medicine, St. Louis, MO 63110 USA; 2 Present Address: Faculty of Medicine, University of Tsukuba, Tsukuba, Ibaraki 305-8575 Japan

## Abstract

Although ubiquitin ligases have been implicated in autism, their roles and mechanisms in brain development remain incompletely understood. Here, we report that in vivo knockdown or conditional knockout of the autism-linked ubiquitin ligase RNF8 or associated ubiquitin-conjugating enzyme UBC13 in rodent cerebellar granule neurons robustly increases the number of parallel fiber presynaptic boutons and functional parallel fiber/Purkinje cell synapses. In contrast to the role of nuclear RNF8 in proliferating cells, RNF8 operates in the cytoplasm in neurons to suppress synapse differentiation in vivo. Proteomics analyses reveal that neuronal RNF8 interacts with the HECT domain protein HERC2 and scaffold protein NEURL4, and knockdown of HERC2 or NEURL4 phenocopies the inhibition of RNF8/UBC13 signaling on synapse differentiation. In behavior analyses, granule neuron-specific knockout of RNF8 or UBC13 impairs cerebellar-dependent learning. Our study defines RNF8 and UBC13 as components of a novel cytoplasmic ubiquitin-signaling network that suppresses synapse formation in the brain.

## Abstract (web summary)

Ubiquitin ligases play critical roles in neuronal connectivity in the brain. Here, Valnegri and colleagues show that ubiquitin ligase RNF8 and ubiquitin-conjugating enzyme UBC13 regulate synapse number in cerebellar granule neurons and rodent cerebellar learning.

## Introduction

Covalent modification of proteins by ubiquitin plays critical roles in the establishment of neuronal connectivity in the developing brain^1, 2^. Protein ubiquitination requires the activity of an E1 ubiquitin-activating enzyme, E2 ubiquitin-conjugating enzyme, and E3 ubiquitin ligase^3, 4^. Ubiquitin ligases are thought to provide specificity in ubiquitin-signaling and may thus play regulatory roles in key steps of neuronal development.

Deregulation of diverse ubiquitin ligases contributes to neurodevelopmental disorders of cognition^5, 6^. For example, mutations in the HECT domain ubiquitin ligase UBE3A cause Angelman syndrome, featuring intellectual disability and autism^7–10^. Genome-wide and homozygosity mapping studies in patients with autism have uncovered copy number variations in the ubiquitin ligases PARK2, RFWD2, and FBXO40^11–13^, a homozygous missense mutation in the HECT domain protein HERC2^14^, and a deletion in the regulatory region of the gene encoding the RING finger ubiquitin ligase RNF8^15^. Whereas the functions and mechanisms of UBE3A and PARK2 are beginning to be elucidated^1, 16–19^, the roles of other autism-linked E3 ubiquitin ligases such as RNF8 in the brain have remained unexplored.

RNF8 has been implicated in DNA damage signaling in proliferating cells^20, 21^. RNF8 is recruited to sites of DNA damage via interaction of its phosphothreonine-binding FHA domain with the adapter protein MDC1, which is phosphorylated by the protein kinase ATM^20, 22^. Once recruited, RNF8 acts with the E2 enzyme UBC13 to catalyze the K63-linked ubiquitination of histone H1^23^. Notably, RNF8 may also interact with the E2 enzyme UBCH8 and promote the K48-linked ubiquitination and turnover of DNA repair proteins^24, 25^. Whereas RNF8 functions have been characterized in DNA damage signaling in proliferating cells, RNF8 functions in the brain have remained unknown.

In this study, we have discovered an RNF8/UBC13 ubiquitin-signaling mechanism that regulates cerebellar synaptic connectivity and motor learning. Knockdown and conditional knockouts of RNF8 and UBC13 in granule neurons in the rodent cerebellum robustly increase the number of functional parallel fiber/Purkinje cell synapses in vivo. Strikingly, structure–function analyses show that RNF8 operates in the cytoplasm in post-mitotic neurons to suppress synapse formation in vivo. Interaction proteomics analyses reveal that RNF8 forms a complex with the autism-linked HECT domain protein HERC2 and scaffold protein Neuralized 4 (NEURL4). Knockdown of HERC2 or NEURL4 mimics the effect of RNF8 inhibition on synapse formation in vivo. In behavior analyses, conditional knockout of RNF8, or UBC13 impairs cerebellar-dependent learning. Our findings define RNF8 and UBC13 as components of a novel cytoplasmic ubiquitin-signaling mechanism in neurons that suppresses synapse formation in the developing brain.

## Results

### RNF8 suppresses synapse formation in the cerebellum

Granule neurons of the cerebellar cortex represent an ideal system for discovery of cell-intrinsic mechanisms that underlie neuronal morphogenesis and connectivity in the mammalian brain^26–28^. Converging evidence in patients and mice suggests that the cerebellum may represent a critical site in the pathogenesis of autism^29–31^. Because deregulation of neuronal connectivity is thought to contribute to autism^32–34^, we assessed the potential role of the autism-linked ubiquitin ligase RNF8 in synapse development in the cerebellum.

RNF8 mRNA was highly expressed in the cerebellum of rat pups during the second and third weeks of postnatal development (Supplementary Fig. 1a), coinciding temporally with synapse formation in the cerebellar cortex. RNF8 protein was expressed in the cerebellum in two- and three-week old rat pups and declined into adulthood (Supplementary Fig. 1b). Notably, RNF8 protein levels were increased in granule neurons upon inhibition of the proteasome (Supplementary Fig. 1c).

To assess the role of RNF8 in synapse development in the rodent cerebellum, we visualized granule neuron presynaptic boutons using an in vivo electroporation method^35–37^ in which we expressed green fluorescent protein (GFP) in granule neurons in rat pups. Rat pups were electroporated at postnatal day 4 (P4) with the GFP expression plasmid, and animals were sacrificed 8 days later at P12. The cerebellum was subjected to immunohistochemistry using the GFP antibody, facilitating visualization of parallel fiber varicosities in the molecular layer of the cerebellar cortex. Parallel fiber GFP-varicosities co-localized with the active zone protein Bassoon and were contiguous with the postsynaptic protein GluR2 (Supplementary Fig. 1d), indicating that parallel fiber GFP-labeled synaptic varicosities represent presynaptic boutons^38^.

**Fig. 1:**
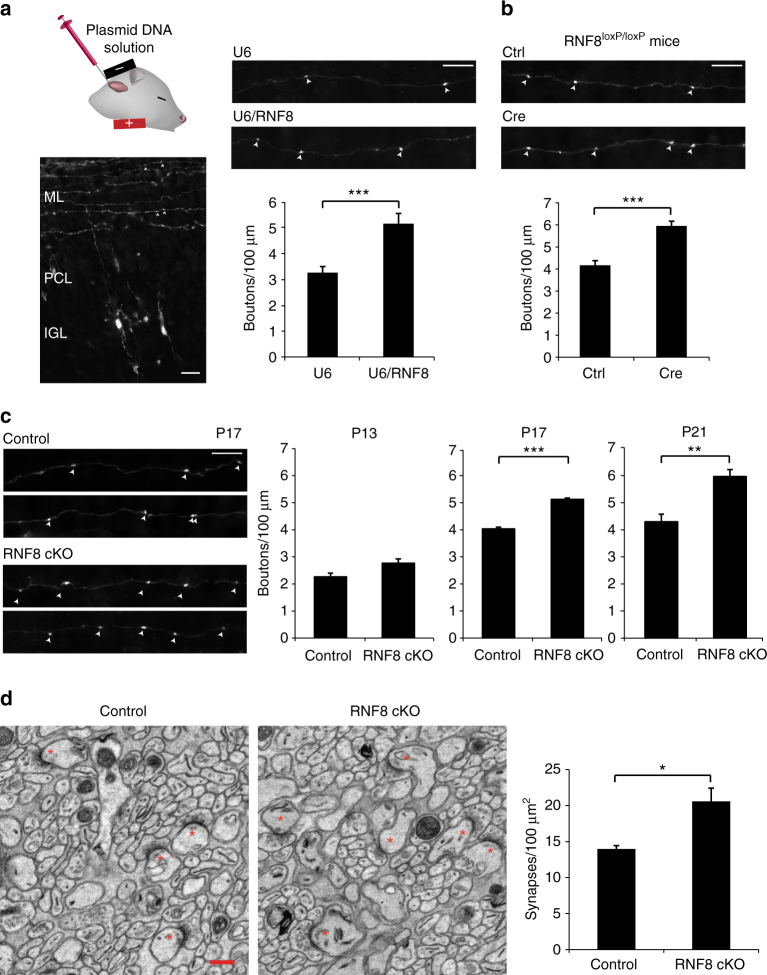
The E3 ubiquitin ligase RNF8 suppresses presynaptic differentiation and synapse formation in the cerebellum in vivo. **a** Left: a representative image of a cerebellum from a P12 rat pup electroporated with the GFP expression plasmid 8 days earlier. Granule neurons have descended to the internal granule layer (IGL) and axonal parallel fibers reside in the molecular layer (ML). Arrowheads denote varicose structures along the parallel fibers that represent presynaptic boutons. Right: P4 rat pups were electroporated with the RNF8 RNAi or control U6 plasmid together with a GFP expression plasmid and sacrificed 8 days later. The cerebellum was removed, sectioned, and subjected to immunohistochemistry using a GFP antibody. Knockdown of RNF8 increased the density of presynaptic boutons in the cerebellar cortex in vivo (***p < 0.05, *t* test, *n* = 5 rats). **b** P9 *RNF8* ^*loxP*/*loxP*^ mice were electroporated with the Cre expression plasmid or control vector together with the GFP expression plasmid and boutons along granule neuron parallel fibers were analyzed as in **a**. Cre-induced knockout of RNF8 in granule neurons increased presynaptic bouton density (***p < 0.001, *t* test, *n* = 6 mice). **c** P9 RNF8 conditional knockout (RNF8 cKO) and control *RNF8* ^*loxP*/*loxP*^ mice were electroporated with the GFP expression plasmid and analyzed at different stages of synapse development as in **a**. Left: representative axons at P17. Right: quantification of the number of presynaptic boutons at different stages of development. Little or no difference in presynaptic bouton number was observed at P13. By contrast, the number of presynaptic boutons was increased at P17 upon conditional knockout of RNF8 (***p < 0.001, *t* test, *n* = 3 mice), and this difference was maintained at P21 (**p < 0.01, *t* test, *n* = 4–5 mice). Scale bars represent 10 μm. **d** The cerebellum from P24 RNF8 cKO and control mice were subjected to electron microscopy analyses. Left: representative electron micrographs of the molecular layer of the cerebellar cortex. Parallel fiber/Purkinje cell synapses are denoted by asterisks. Scale bar = 500 nm. Right: quantification of the density of parallel fiber/Purkinje cell synapses in RNF8 cKO and control mice. The density of parallel fiber/Purkinje cell synapses was increased in RNF8 cKO mice compared to control mice (*p < 0.05, *t* test, *n* = 3 mice)

We induced the knockdown of RNF8 using a plasmid-based method of RNAi^39, 40^. Expression of RNF8 shRNAs led to knockdown of endogenous RNF8 in primary granule neurons (U6/RNF8: 46.89 ± 7.98% of control, *n* = 3 cultures; mean ± s.e.m.; *p* = 0.003, *t* test; U6/RNF8.2: 26.62 ± 5.72% of control, *n* = 3 cultures; mean ± s.e.m.; *p* = 0.0002, *t* test) (Supplementary Fig. 1e). Knockdown of RNF8 robustly increased the number of parallel fiber presynaptic boutons in vivo (U6: 3.255 ± 0.235 boutons/100 μm; U6/RNF8: 5.155 ± 0.392 boutons/100 μm; *n* = 5 rats; mean ± s.e.m.; *p* = 3.556E−05, *t* test) (Fig. 1a), (U6: 3.893 ± 0.371 boutons/100 μm; U6/RNF8.2: 6.758 ± 0.589 boutons/100 μm; *n* = 3 rats; mean ± s.e.m.; *p* = 0.0183, *t* test) (Supplementary Fig. 1f). These results suggest that RNF8 suppresses the formation of granule neuron presynaptic boutons.

We next employed a conditional mouse genetics approach in which loxP sites were placed flanking exons 6 and 7 of the *RNF8* gene (Supplementary Fig. 2a). Using the in vivo electroporation approach to express the recombinase Cre, induction of RNF8 knockout in granule neurons significantly increased the number of parallel fiber presynaptic boutons (Ctrl: 4.164 ± 0.206 boutons/100 μm; Cre: 5.943 ± 0.213 boutons/100 μm; *n* = 6 mice; mean ± s.e.m.; *p* = 0.0001, *t* test) (Fig. 1b).

We also crossed mice harboring the floxed allele of the *RNF8* gene with mice in which the Cre driver is controlled by the promoter of the *Math1* gene, which is expressed in granule cells within the cerebellar cortex^41^. Mice in which the *RNF8* gene was knocked out using *Math1-Cre* were viable and weighed similarly to control littermate animals (Supplementary Fig. 2b). Notably, global RNF8 knockout mice^42^ were smaller than control littermate mice (data not shown). Conditional knockout of RNF8 reduced the abundance of RNF8 mRNA in the cerebellum of P10 mice by ~60% (RNF8 cKO: 44.88 ± 1.06% of control, *n* = 3 mice; *p* = 8.274E−07, *t* test) (Supplementary Fig. 2c, d). Global RNF8 knockout downregulated the abundance of RNF8 mRNA in the cerebellum by almost 100% (Supplementary Fig. 2d). Because granule neurons represent 70% of the cells in the cerebellum^43^, these results suggest that *Math1-Cre* leads to efficient knockout of RNF8 in granule neurons of the cerebellum. No anatomical abnormalities were evident in the cerebellum of RNF8 conditional knockout mice (Supplementary Fig. 2e), suggesting that conditional knockout of RNF8 specifically alters presynaptic bouton differentiation.

We next characterized presynaptic differentiation in RNF8 conditional knockout mice at different stages of synapse development. There was little or no difference in parallel fiber presynaptic bouton number at P13 (control: 2.25 ± 0.138 boutons/100 μm; RNF8 cKO: 2.76 ± 0.156 boutons/100 μm; *n* = 4 mice; mean ± s.e.m.; *p* = 0.0503, *t* test) (Fig. 1c). By contrast, the number of parallel fiber presynaptic boutons was increased at P17 upon conditional knockout of RNF8 (control: 4.07 ± 0.065 boutons/100 μm; RNF8 cKO: 5.16 ± 0.051 boutons/100 μm; *n* = 3 mice; mean ± s.e.m.; *p* = 8.27E−05, *t* test), and this difference was maintained at P21 (control: 4.28 ± 0.283 boutons/100 μm; RNF8 cKO: 5.94 ± 0.243 boutons/100 μm; *n* = 4–5 mice; mean ± s.e.m.; *p* = 0.003, *t* test) (Fig. 1c).

In corroborating electron microscopy (EM) analyses, the number of parallel fiber/Purkinje cell synapses was significantly increased in RNF8 conditional knockout mice compared to control littermate mice (control: 13.98 ± 0.469 synapses/100 μm^2^; RNF8 cKO: 20.59 ± 1.843 synapses/100 μm^2^; *n* = 3 mice; mean ± s.e.m.; *p* = 0.025, *t* test) (Fig. 1d). Notably, parallel fiber/Purkinje cell synapse number was also increased in global RNF8 knockout mice (data not shown). Collectively, our results establish that RNF8 suppresses formation of parallel fiber/Purkinje cell synapses in the developing cerebellum.

### RNF8 suppresses granule neuron/Purkinje cell transmission

**Fig. 2:**
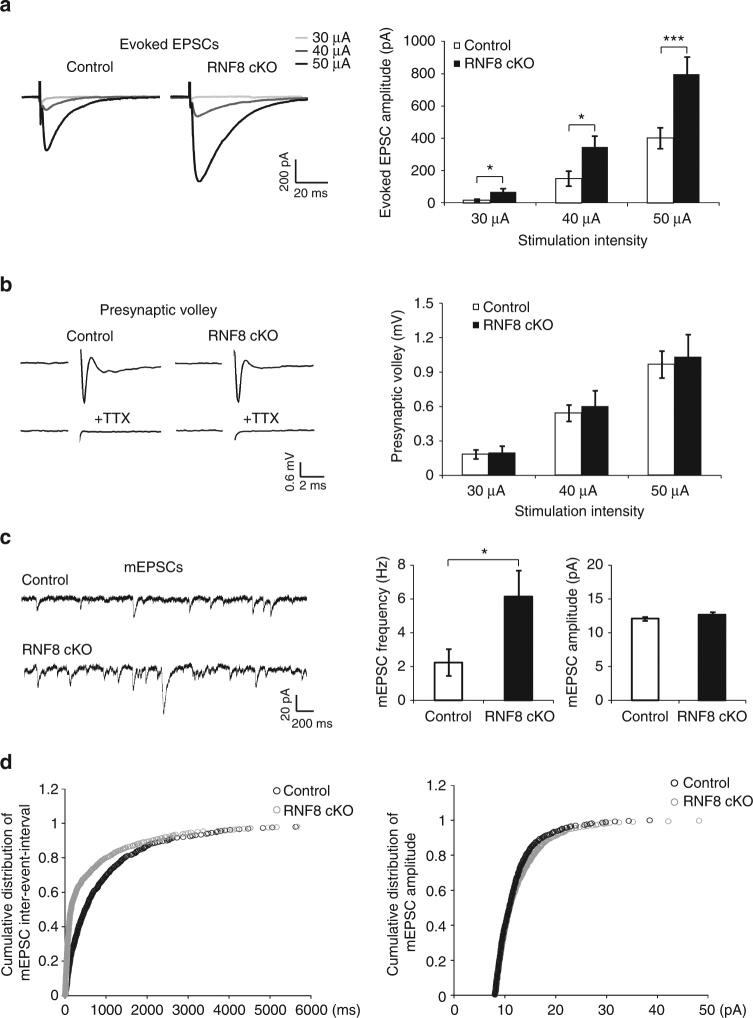
RNF8 suppresses granule neuron to Purkinje cell neurotransmission in the cerebellum. **a** Acute sagittal cerebellar slices from P20–25 RNF8 cKO and control mice were subjected to electrophysiological analyses. Evoked excitatory postsynaptic currents (EPSCs) were recorded in Purkinje neurons in response to stimulation of parallel fibers with increasing intensity (30, 40, and 50 μA). Representative current traces (left) and quantification of the amplitude of evoked EPSCs (right) are shown. The amplitude of evoked EPSCs in Purkinje neurons was increased in RNF8 cKO mice compared to control mice (*p < 0.05 at 30 and 40 μA, ***p < 0.005 at 50 μA, ANOVA followed by Fisher’s PLSD post hoc test, *n* = 14–15 neurons, four mice). **b** Acute coronal cerebellar slices were prepared as in (**a**), and parallel fiber axons were stimulated at sites 400 μm away from an extracellular recording electrode. A representative trace of the stimulus-evoked presynaptic waveform before and after the application of tetrodotoxin is shown (left). The stimulus artifact was removed for clarity. On the right, quantification of presynaptic volley amplitude is shown. Conditional knockout of RNF8 had little or no effect on the presynaptic volley amplitude. **c**, **d** Acute sagittal cerebellar slices were prepared as in (**a**) and Purkinje cell miniature EPSCs (mEPSCs) were recorded. Representative traces of mEPSCs from RNF8 cKO and control mice are shown (**c**, left). Quantification of the mean (**c**, right) and cumulative distribution (**d**) of the mEPSC frequency and amplitude are shown. The frequency of mEPSCs was increased in RNF8 cKO mice compared to control mice (*p < 0.05, *t* test, *n* = 18–20 neurons, three mice). Conditional knockout of RNF8 had little or no effect on the amplitude of mEPSCs in Purkinje neurons

We next assessed the effect of conditional knockout of RNF8 on neurotransmission at parallel fiber/Purkinje cell synapses. Electrophysiological analyses in acute cerebellar slices revealed that the amplitude of evoked excitatory postsynaptic currents (EPSCs) at parallel fiber/Purkinje cell synapses was increased in RNF8 conditional knockout mice compared to control animals (at 30 μA, control: 15.18 ± 7.13 pA; RNF8 cKO: 67 ± 23.33 pA; *p* = 0.039; at 40 μA, control: 151.27 ± 47.42 pA; RNF8 cKO: 348.64 ± 65.67 pA; *p* = 0.021; at 50 μA, control: 402 ± 67.71 pA; RNF8 cKO: 797.86 ± 107.93 pA; *p* = 0.004; *n* = 14–15 neurons, four mice; mean ± s.e.m.; ANOVA followed by Fisher’s protected least significant difference (PLSD) post hoc test) (Fig. 2a). In control analyses, there was little or no difference in the amplitude of presynaptic volleys in control and RNF8 conditional knockout mice (at 30 μA, control: 0.187 ± 0.038 mV; RNF8 cKO: 0.199 ± 0.059 mV; *p* = 0.991; at 40 μA, control: 0.544 ± 0.072 mV; RNF8 cKO: 0.604 ± 0.139 mV; *p* = 0.746; at 50 μA, control: 0.966 ± 0.118 mV; RNF8 cKO: 1.036 ± 0.195 mV; *p* = 0.916; *n* = 13–17 field recordings, two mice; mean ± s.e.m.; ANOVA followed by Fisher’s PLSD post hoc test) (Fig. 2b), suggesting that the increased amplitude of evoked EPSCs in RNF8 conditional knockout mice reflects alterations in synaptic function rather than changes in axon excitability.

We next asked if augmented neurotransmission in RNF8 conditional knockout mice might be secondary to changes in synapse number. The frequency of miniature EPSCs (mEPSCs) in Purkinje cells was increased in acute cerebellar slices from RNF8 conditional knockout mice compared to control animals (control: 2.26 ± 0.8 Hz; RNF8 cKO: 6.14 ± 1.58 Hz; *n* = 18–20 neurons, three mice; mean ± s.e.m.; *p* = 0.041, *t* test of means; *p* < 0.001, Kolmogorov–Smirnov test of cumulative distribution) (Fig. 2c, d), consistent with the conclusion that synapse number is increased in RNF8 conditional knockout mice. There was little or no change in the amplitude of mEPSCs in RNF8 conditional knockout mice (control: 12.15 ± 0.27 pA; RNF8 cKO: 12.68 ± 0.43 pA; *n* = 18–20 neurons, three mice; mean ± s.e.m.; *p* = 0.315, *t* test of means; *p* = 0.43, Kolmogorov–Smirnov test of cumulative distribution) (Fig. 2c, d). There was little or no difference in the rise and decay time of mEPSCs in control and RNF8 conditional knockout mice (Supplementary Fig. 3 and Supplementary Table 1).

In analyses of granule neuron intrinsic excitability, there was little or no difference in the number of evoked action potentials (AP; at 10pA, control: 0.7 ± 0.517; RNF8 cKO: 1 ± 1; *p* = 0.793; at 20pA, control: 6.6 ± 3.008; RNF8 cKO: 6.7 ± 3.222; *p* = 0.982; at 30pA, control: 13.4 ± 3.945; RNF8 cKO: 13.2 ± 4.948; *p* = 0.975; at 40 pA, control: 21 ± 4.198; RNF8cKO: 16.7 ± 6.763; *p* = 0.61; *n* = 10 neurons, two mice; mean ± s.e.m.; ANOVA followed by Fisher’s PLSD post hoc test), AP amplitude (control: 36.444 ± 1.973 mV; RNF8 cKO: 38.511 ± 3.21 mV; *n* = 9 neurons, two mice; mean ± s.e.m.; *p* = 0.591, *t* test), and input resistance (control: 1.418 ± 0.059 GΩ; RNF8 cKO: 1.386 ± 0.088 GΩ; *n* = 10 neurons, two mice; mean ± s.e.m.; *p* = 0.765, *t* test) in control and RNF8 conditional knockout mice (Supplementary Fig. 4a, b). These data suggest that RNF8 may not set the intrinsic excitability of granule neurons. In other analyses, little or no difference in paired-pulse facilitation was found in control and RNF8 conditional knockout mice (control: 2.55 ± 0.149; RNF8 cKO: 2.47 ± 0.233; *n* = 8 neurons, three mice; mean ± s.e.m.; *p* = 0.771, *t* test) (Supplementary Fig. 4c), suggesting RNF8 may not control the probability of vesicle release at parallel fiber/Purkinje cell synapses in the mouse cerebellum. Taken together, our findings suggest that RNF8 influences the structural and functional maturation of synapses in the cerebellar cortex.

### RNF8 operates in the cytoplasm to suppress synaptogenesis

**Fig. 3:**
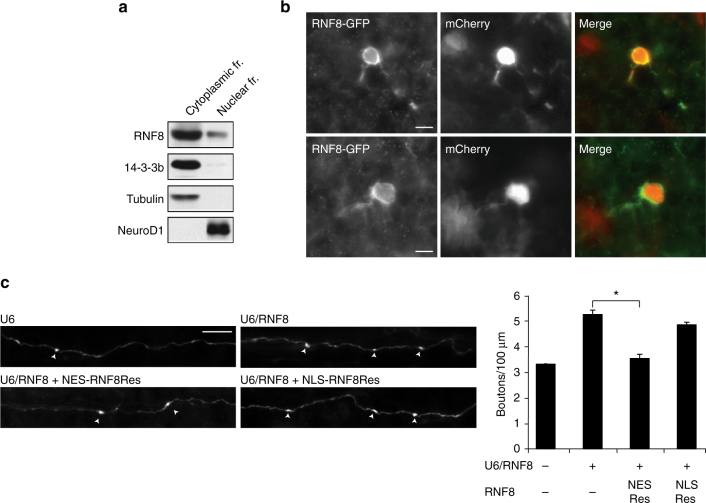
RNF8 operates in the cytoplasm to suppress presynaptic differentiation in vivo. **a** Immunoblotting analyses of fractionated lysates of P8 rat cerebellum revealed abundant endogenous RNF8 in the cytoplasmic fraction in the cerebellum. **b** P4 rat pups were electroporated with an expression plasmid encoding an RNF8–GFP fusion protein together with a mCherry expression plasmid. After 8 days, rat pups were sacrificed and the cerebellum was subjected to immunohistochemistry using GFP and DsRed (mCherry) antibodies. RNF8 localizes predominantly in the cytoplasm. **c** P4 rat pups were electroporated with the control U6 plasmid or RNF8 RNAi plasmid together with an expression plasmid encoding NES(nuclear export signal)-RNF8Res fusion protein, NLS(nuclear localization signal)-RNF8Res fusion protein or the control vector and with the GFP expression plasmid and analyzed as in Fig. 1a. Expression of NES-RNF8Res, but not NLS-RNF8Res, reversed the RNF8 RNAi-induced phenotype of increased number of parallel fiber presynaptic boutons in the cerebellum in vivo (*p < 0.05, ANOVA followed by Fisher’s PLSD post hoc test, *n* = 3–4 rats). Scale bars = 10 μm

We next determined the mechanism underlying RNF8 function in the brain. The subcellular site of ubiquitin ligases plays a critical role in specifying their functions in neurons^1, 44–46^. RNF8 acts in the nucleus to mediate cellular responses to DNA damage in proliferating cells^20, 25^. Surprisingly, in immunoblotting analyses of fractionated lysates of granule neurons, RNF8 immunoreactivity was present primarily in the cytoplasm in neurons, though RNF8 was also present in the nucleus (Fig. 3a). Antibodies to RNF8 performed poorly in immunocytochemical and immunohistochemical analyses. We therefore characterized the localization of an RNF8–GFP fusion protein in granule neurons in the cerebellum. RNF8–GFP was localized predominantly in the cytoplasm in granule neurons in vivo (Fig. 3b).

We next performed structure–function analyses of RNF8 function in the control of synapse development in the background of RNF8 RNAi in rodent pups. We used the in vivo electroporation approach in rodent pups to target RNF8 to the cytoplasm or nucleus in granule neurons by appending a nuclear export signal (NES) or nuclear localization signal (NLS), respectively, to an RNAi-resistant rescue form of RNF8 (RNF8Res) (Supplementary Fig. 5a, b). Expression of NES-RNF8Res, but not NLS-RNF8Res, reversed the RNF8 RNAi-induced phenotype of increased parallel fiber presynaptic bouton number in vivo (U6: 3.352 ± 0.153 boutons/100 μm; U6/RNF8: 5.192 ± 0.478 boutons/100 μm; U6/RNF8 + NES Res: 3.535 ± 0.162 boutons/100 μm; U6/RNF8 + NLS Res: 4.854 ± 0.099 boutons/100 μm; *n* = 3–4 rats; mean ± s.e.m.; U6/RNF8 + NES Res vs. U6/RNF8: *p* = 0.013; U6/RNF8 + NLS Res vs. U6/RNF8: *p* = 0.527, ANOVA followed by Fisher’s PLSD post hoc test) (Fig. 3c). The exogenous expression of RNF8 in the absence of RNF8 RNAi had little or no effect on the number of parallel fiber boutons in vivo (GFP: 4.03 ± 0.223 boutons/100 μm; GFP + RNF8: 4.011 ± 0.171 boutons/100 μm; *n* = 4 rats; mean ± s.e.m.; *p* = 0.95, *t* test) (Supplementary Fig. 5c). Together, our results suggest that RNF8 operates in the cytoplasm rather than the nucleus in post-mitotic neurons to suppress synapse differentiation.

### UBC13 acts with RNF8 to suppress synapse differentiation

**Fig. 4:**
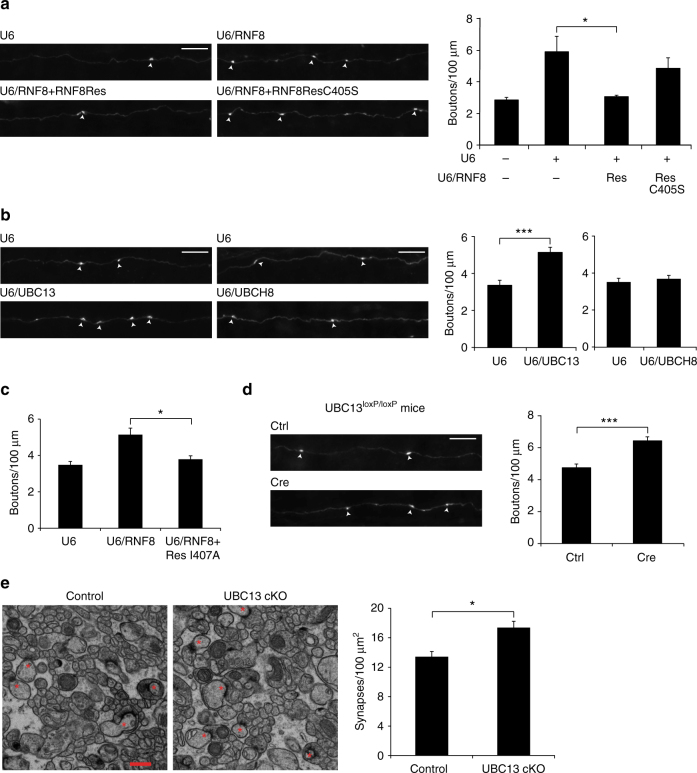
The ubiquitin-conjugating E2 enzyme UBC13 operates with RNF8 to suppress presynaptic differentiation in vivo. **a** P4 rat pups were electroporated with the control U6 plasmid or RNF8 RNAi plasmid together with an expression plasmid encoding RNF8Res, RNF8Res in which C405 was mutated to serine (RNF8ResC405S), or the control vector and with the GFP expression plasmid and analyzed as in Fig. 1a. RNF8Res, but not RNF8ResC405S, reversed the RNF8 RNAi-induced phenotype of increased presynaptic bouton number in vivo (*p < 0.05, ANOVA followed by Fisher’s PLSD post hoc test, *n* = 3-4 rats). **b** P4 rat pups were electroporated with the UBC13 RNAi, UBCH8 RNAi, or control U6 plasmid and analyzed as in Fig. 1a. Knockdown of UBC13, but not knockdown of UBCH8, increased the density of presynaptic parallel fiber boutons in the cerebellar cortex in vivo (***p < 0.005, *t* test, *n* = 3 rats). **c** P4 rat pups were electroporated with the control U6 plasmid or RNF8 RNAi together with a RNF8Res mutant in which isoleucine 407 was replaced with alanine (RNF8ResI407A) or the control vector and with the GFP expression plasmid and analyzed as in Fig. 1a. Expression of RNF8ResI407A reversed the RNF8 knockdown-induced phenotype on presynaptic boutons (*p < 0.05, ANOVA followed by Fisher’s PLSD post hoc test, *n* = 3–6 rats). For representative images of axons see Supplementary Fig. 6c. **d** P6/7 *UBC13* ^*loxP*/*loxP*^ mice were electroporated with the Cre expression plasmid or control vector together with the GFP expression plasmid and analyzed as in Fig. 1a. Cre-induced knockout of UBC13 in granule neurons increased presynaptic bouton density in vivo (***p < 0.001, *t* test, *n* = 6 mice). Scale bars = 10 μm. **e** The cerebellum from P25 UBC13 conditional knockout mice and control *UBC13* ^*loxP*/*loxP*^ mice were subjected to electron microscopy analyses. Left: representative electron micrographs of the molecular layer of the cerebellar cortex. Parallel fiber presynaptic bouton/Purkinje cell synapses are denoted by asterisks. Scale bar = 500 nm. Right: quantification of the density of granule neuron parallel fiber/Purkinje cell synapses in UBC13 cKO and control mice. Knockout of UBC13 induced an increase in the density of parallel fiber/Purkinje cell synapses compared to control mice (*p < 0.05, *t* test, *n* = 3 mice)

The finding that RNF8 acts in the cytoplasm rather than the nucleus in neurons led us next to the question of whether the ubiquitin ligase activity of RNF8 is required for its function in neurons. Expression of RNF8Res effectively reversed the RNF8 RNAi-induced phenotype of increased parallel fiber presynaptic bouton number in the cerebellum in vivo (U6: 2.848 ± 0.158 boutons/100 μm; U6/RNF8: 5.892 ± 0.982 boutons/100 μm; U6/RNF8 + Res: 3.033 ± 0.09 boutons/100 μm; U6/RNF8 + ResC405S: 4.825 ± 0.666 boutons/100 μm; *n* = 3–4 rats; mean ± s.e.m.; U6/RNF8 + Res vs. U6/RNF8: *p* = 0.044; ANOVA followed by Fisher’s PLSD post hoc test) (Fig. 4a). By contrast, expression of a mutant form of RNF8Res in which the key regulatory RING domain site cysteine 405 was replaced with serine (RNF8ResC405S) failed to effectively reverse the RNF8 RNAi-induced presynaptic bouton phenotype (U6/RNF8 + ResC405S vs U6/RNF8: *p* = 0.42; ANOVA followed by Fisher’s PLSD post hoc test) (Fig. 4a). These results suggest that the ubiquitin ligase activity of RNF8 suppresses synapse differentiation.

RNF8 interacts with the ubiquitin-conjugating enzyme UBC13 or UBCH8, and thereby catalyzes K63-linked or K48-linked ubiquitin chains, respectively^21, 24, 47, 48^. We asked whether RNF8 acts via UBC13 or UBCH8 to suppress synapse differentiation. Knockdown of UBC13, but not knockdown of UBCH8, robustly increased the number of parallel fiber presynaptic boutons in vivo (U6: 3.28 ± 0.175 boutons/100 μm; U6/UBC13: 5.15 ± 0.269 boutons/100 μm; *n* = 3 rats; mean ± s.e.m.; *p* = 0.004, *t* test; U6: 3.51 ± 0.205 boutons/100 μm; U6/UBCH8: 3.69 ± 0.184 boutons/100 μm; *n* = 3 rats; mean ± s.e.m.; *p* = 0.565, *t* test) (Fig. 4b and Supplementary Fig. 6a, b). Consistent with these results, expression of a mutant form of RNF8Res (RNF8ResI407A), which fails to interact with UBCH8 but maintains interaction with UBC13^24^, reversed effectively the RNF8 knockdown-induced phenotype of increased parallel fiber presynaptic bouton number in vivo (U6: 3.48 ± 0.19 boutons/100 μm; U6/RNF8: 5.147 ± 0.358 boutons/100 μm; U6/RNF8 + ResI407A: 3.79 ± 0.19 boutons/100 μm; *n* = 3–6 rats; mean ± s.e.m.; U6/RNF8 + ResI407A vs. U6/RNF8: *p* = 0.027; ANOVA followed by Fisher’s PLSD post hoc test) (Fig. 4c and Supplementary Fig. 6c). Together, these results suggest UBC13, but not UBCH8, operates with RNF8 to regulate synapse development.

In the in vivo electroporation approach to express Cre in granule neurons in mice that harbor a floxed allele of the *UBC13* gene (*UBC13* ^*loxP*/*loxP*^), conditional knockout of UBC13 significantly increased the number of parallel fiber presynaptic boutons (control: 4.75 ± 0.23 boutons/100 μm; Cre: 6.44 ± 0.247 boutons/100 μm; *n* = 6 mice; mean ± s.e.m.; *p* = 0.0005, *t* test) (Fig. 4d). Conditional knockout of UBC13 had little or no effect on the number of parallel fiber presynaptic boutons at P13 (control: 2.73 ± 0.135 boutons/100 μm; UBC13 Cre: 3.16 ± 0.194 boutons/100 μm; *n* = 3–4 mice; *p* = 0.148; mean ± s.e.m., *t* test) (Supplementary Fig. 7a). By contrast, conditional knockout of UBC13 significantly increased parallel fiber presynaptic bouton number at P17 (control: 3.94 ± 0.208 boutons/100 μm; UBC13 Cre: 5.37 ± 0.311 boutons/100 μm; *n* = 3 mice; *p* = 0.019; mean ± s.e.m., *t* test), and this difference was maintained at P21 (control: 4.21 ± 0.203 boutons/100 μm; UBC13 Cre: 5.96 ± 0.084 boutons/100 μm; *n* = 3 mice; *p* = 0.005; mean ± s.e.m., *t* test) (Supplementary Fig. 7a). Together, these data suggest that UBC13 suppresses the differentiation of presynaptic boutons.

We next crossed *UBC13* ^*loxP*/*loxP*^ mice with mice in which Cre is expressed using the granule neuron-specific driver *GABAα6* receptor gene promoter^49^. UBC13 protein and mRNA levels in the cerebellum were downregulated in granule neuron-specific UBC13 conditional knockout mice (Supplementary Fig. 7b, c). UBC13 conditional knockout mice were viable and weighed similarly  to control littermate animals (Supplementary Fig. 7d), and had little or no alterations in the overall architecture of the cerebellar cortex (Supplementary Fig. 7e). EM analyses revealed an increased number of parallel fiber/Purkinje cell synapses in the cerebellar cortex in UBC13 conditional knockout mice (control: 13.43 ± 0.709 synapses/100 μm^2^; UBC13 cKO: 17.38 ± 0.867 synapses/100 μm^2^; *n* = 3 mice; mean ± s.e.m.; *p* = 0.024, *t* test) (Fig. 4e), suggesting that UBC13 suppresses synapse formation in the cerebellar cortex.

**Fig. 5:**
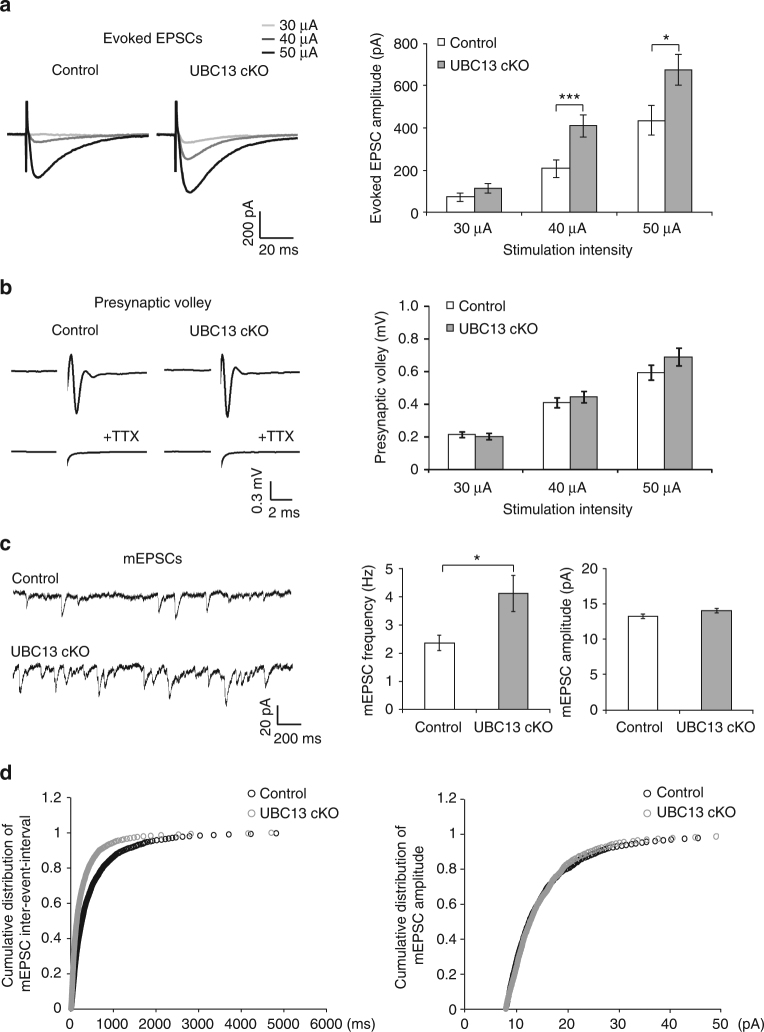
UBC13 suppresses granule neuron to Purkinje cell neurotransmission in the cerebellum. **a** Acute sagittal cerebellar slices from P23–26 UBC13 cKO and control mice were subjected to electrophysiological analyses. Evoked excitatory postsynaptic currents (EPSCs) were recorded in Purkinje neurons in response to stimulation of parallel fibers with increasing intensity (30, 40, and 50 μA). Representative current traces (left) and quantification of the amplitude of evoked EPSCs (right) are shown. The amplitude of evoked EPSCs in Purkinje neurons was increased in UBC13 cKO mice compared to control mice (***p < 0.005 at 40 μA, *p < 0.05 at 50 μA, ANOVA followed by Fisher’s PLSD post hoc test, *n* = 25–29, six mice). **b** Acute coronal cerebellar slices were prepared as in (**a**), and parallel fiber axons were stimulated at sites 400 μm away from an extracellular recording electrode. A representative trace of the stimulus-evoked presynaptic waveform before and after the application of tetrodotoxin is shown (left). The stimulus artifact was removed for clarity. On the right, quantification of presynaptic volley amplitude is shown. Conditional knockout of UBC13 had little or no effect on the presynaptic volley amplitude. **c**, **d** Acute sagittal cerebellar slices were prepared as in (**a**) and Purkinje cell mEPSCs were recorded. Representative traces of mEPSCs from UBC13 cKO mice and control mice are shown (**c**, left). Quantification of the mean (**c**, right) and cumulative distribution (**d**) of the mEPSC frequency and amplitude are shown. The frequency of mEPSCs was increased in UBC13 cKO mice compared to control mice (*p < 0.05, *t* test, *n* = 32 neurons, six mice). Conditional knockout of UBC13 had little or no effect on the amplitude of mEPSCs in Purkinje neurons

In electrophysiological analyses in acute cerebellar slices, the amplitude of evoked EPSCs at parallel fiber/Purkinje cell synapses was substantially increased in UBC13 conditional knockout mice compared to control animals (at 30 μA, control: 71.26 ± 20.23 pA; UBC13 cKO: 113.72 ± 23.03 pA; *p* = 0.167; at 40 μA, control: 206.41 ± 42.03 pA; UBC13 cKO: 408.62 ± 52.26 pA; *p* = 0.003; at 50 μA, control: 434.41 ± 69.22 pA; UBC13 cKO: 675.62 ± 73.38 pA; *p* = 0.019; *n* = 25–29 neurons, six mice; mean ± s.e.m.; ANOVA followed by Fisher’s PLSD post hoc test) (Fig. 5a). There was little or no difference in the amplitude of presynaptic volleys in control and UBC13 conditional knockout mice (at 30 μA, control: 0.212 ± 0.017 mV; UBC13 cKO: 0.202 ± 0.019 mV; *p* = 0.69; at 40 μA, control: 0.408 ± 0.03 mV; UBC13 cKO: 0.44 ± 0.035 mV; *p* = 0.45; at 50 μA, control: 0.59 ± 0.047 mV; UBC13 cKO: 0.69 ± 0.055 mV; *p* = 0.19; *n* = 17–20 field recordings, three mice; mean ± s.e.m.; ANOVA followed by Fisher’s PLSD post hoc test) (Fig. 5b), suggesting that the increased amplitude of evoked EPSCs in UBC13 conditional knockout mice is not secondary to changes in axon excitability and instead reflects alterations in synaptic function. In other analyses, the frequency of mEPSCs in Purkinje cells was increased upon conditional knockout of UBC13 mice, consistent with the conclusion that synapse number is increased in UBC13 conditional knockout mice (control: 2.35 ± 0.27 Hz; UBC13 cKO: 4.11 ± 0.64 Hz; *n* = 32 neurons; six mice; mean ± s.e.m.; *p* = 0.013, *t* test of means; *p* < 0.001, Kolmogorov–Smirnov test of cumulative distribution) (Fig. 5c, d). There was little or no change in the amplitude of mEPSCs in UBC13 conditional knockout mice (control: 13.23 ± 0.28 pA; UBC13 cKO: 14.02 ± 0.31 pA; *n* = 32 neurons; six mice; mean ± s.e.m.; *p* = 0.065, *t* test of means; *p* = 0.97, Kolmogorov–Smirnov test of cumulative distribution) (Fig. 5c, d). In addition, conditional knockout of UBC13 led to little or no difference in the rise time of mEPSCs and to a modest increase in the decay time of mEPSCs (Supplementary Fig. 8 and Supplementary Table 2). Together, these data reveal that the structural alterations in parallel fiber/Purkinje cell synapse development in UBC13 conditional knockout mice bear functional consequences on synaptic neurotransmission. Collectively, our findings support the conclusion that UBC13 operates in concert with RNF8 to suppress synapse differentiation in the mammalian brain.

### RNF8-interactors HERC2 and NEURL4 suppress synaptogenesis

**Fig. 6:**
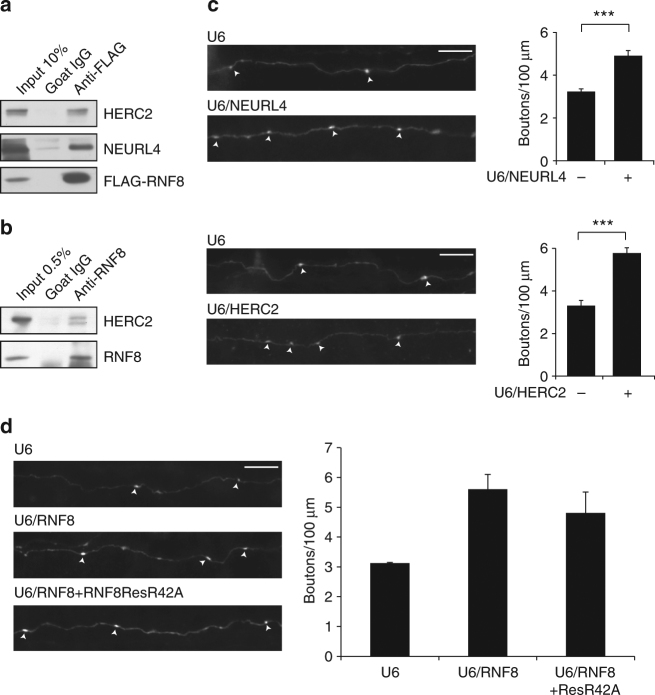
RNF8 interacts with HERC2 and NEURL4 in the cerebellum and thereby suppresses parallel fiber presynaptic differentiation in vivo. **a** Lysates of Neuro2A mouse neuroblastoma cells constitutively expressing FLAG-RNF8 were subjected to immunoprecipitation followed by immunoblotting. Coimmunoprecipitation of FLAG-RNF8 with endogenous HERC2 and NEURL4. **b** The cytoplasmic fraction of lysates of P7 rat cerebellum was subjected to immunoprecipitation followed by immunoblotting. Endogenous coimmunoprecipitation of RNF8 and HERC2 in the cytoplasmic fraction of the cerebellum. **c** P4 rat pups were electroporated with the NEURL4 RNAi, HERC2 RNAi, or control U6 plasmid and analyzed as in Fig. 1a. Knockdown of NEURL4 and HERC2 increased the density of presynaptic parallel fiber boutons in the cerebellum in vivo (NEURL4 RNAi, ***p < 0.005, *t* test, *n* = 3–8 rats; HERC2 RNAi, ***p < 0.001, *t* test, *n* = 6–9 rats). **d** P4 rat pups were electroporated with the control U6 plasmid or RNF8 RNAi together with an RNF8Res mutant in which arginine 42 was replaced with alanine (RNF8ResR42A) or the control vector and with the GFP expression plasmid and analyzed as in Fig. 1a. Expression of RNF8ResR42A failed to reverse the RNF8 knockdown-induced presynaptic bouton phenotype in the cerebellum in vivo. Scale bars = 10 μm

To gain insights into the mechanisms of RNF8/UBC13 signaling in synapse development, we employed a computer-assisted interaction proteomics approach^46, 50–52^. We immunoprecipitated FLAG-RNF8 in Neuro2A mouse neuroblastoma cells followed by mass spectrometry (IP-MS). Eluted trypsinized FLAG-RNF8 bait complexes were subjected to liquid chromatography–MS/MS. We used Neuro2A cells as the neural proteome for the immunoprecipitation of a large number of proteins^52^. This approach facilitates identification of high-confidence interacting proteins using the statistical software Com*PASS* ^50^, which compares the interactome of a specific protein to the interactomes of all other prey proteins in the same cell type. These analyses led to the identification of RNF8-interacting proteins including the HECT domain protein HERC2 and scaffold protein NEURL4, which form a complex^53–55^ (Table 1). Notably, HERC2 plays a critical role in DNA damage signaling^21^. Upon phosphorylation by the protein kinase ATM, HERC2 interacts with RNF8 via the FHA domain of RNF8^21^. HERC2 also interacts with UBC13 and facilitates formation of the RNF8/UBC13 complex that triggers K63-linked ubiquitination^21^. In coimmunoprecipitation analyses in Neuro2A cells in which we expressed RNF8, endogenous HERC2 and NEURL4 interacted with RNF8 (Fig. 6a). HERC2 and NEURL4 mRNA and protein were expressed endogenously in the developing rodent cerebellum (Supplementary Fig. 1a, b). Like RNF8, NEURL4, and HERC2 localized in the cytoplasm in granule neurons (Supplementary Fig. 9a, b). Importantly, endogenous RNF8 interacted with HERC2 in the cytoplasmic fraction of the rodent cerebellum (Fig. 6b).

**Table 1:** RNF8-interacting proteins

Symbol	NWD-score	TSC
Herc2	11.7	107
mouse_Rnf8	9.8	6
Neurl4	6.04	25
Anks1	2.83	2
6330503K22R	2	1
Gle1	2	1
Cep170	1.71	13
Ktn1	1.16	10
Ssbp1	0.98	6
Gm16517	0.8	1

Lysates from Neuro2A mouse neuroblastoma cells expressing FLAG-RNF8 were immunoprecipitated using a resin conjugated with antibodies to FLAG (FLAG M2 agarose) and analyzed by mass spectrometry. Resulting peptides were subjected to computational analyses using the Com*PASS* software IP-MS databases in Neuro2A cells^61^. High-confidence interacting proteins (HCIP) had normalized weighted D score more than 1. Total Spectra Count (TSC) of HCIPs RNF8 is also shown

We next determined the effect of NEURL4 knockdown or HERC2 knockdown on presynaptic bouton formation in the rodent cerebellum. We found that the number of presynaptic boutons was significantly increased in NEURL4 knockdown animals (U6: 3.08 ± 0.133 boutons/100 μm; U6/NEURL4: 4.91 ± 0.274 boutons/100 μm; *n* = 3–8 rats; mean ± s.e.m.; *p* = 0.0016, *t* test) and in HERC2 knockdown animals (U6: 3.29 ± 0.244 boutons/100 μm; U6/HERC2: 5.76 ± 0.246 boutons/100 μm; *n* = 6–9 rats; mean ± s.e.m.; *p* = 3.556E−05, *t* test) (Fig. 6c and Supplementary Fig. 9c, d).

We assessed whether interaction with HERC2 and NEURL4 contributes to the ability of RNF8 to regulate synapse development. Mutation of the FHA domain in RNF8 (R42A) impaired the ability of RNF8 to form a complex with HERC2 and NEURL4 in cells (Supplementary Fig. 9e, f), suggesting that the FHA domain mediates the interaction of RNF8 with HERC2 and NEURL4. In structure–function analyses, an RNF8Res protein in which the FHA domain was mutated (RNF8ResR42A)^20^ failed to effectively reverse the RNF8 knockdown-induced increase in the number of granule neuron parallel fiber presynaptic boutons in vivo (U6: 3.07 ± 0.024 boutons/100 μm; U6/RNF8: 5.604 ± 0.496 boutons/100 μm; U6/RNF8 + ResR42A: 4.878 ± 0.642 boutons/100 μm; *n* = 3 rats; mean ± s.e.m.; U6/RNF8 + ResR42A vs. U6/RNF8: *p* = 0.421; ANOVA followed by Fisher’s PLSD post hoc test) (Fig. 6d). Taken together, these results suggest that the interaction of RNF8 with HERC2 and NEURL4 plays a critical role in the regulation of synapse development in the brain.

### Role of RNF8/UBC13 ubiquitin signaling in motor learning

Alterations of parallel fiber/Purkinje cell synapse number and function have been associated with deficits in cerebellar-dependent procedural motor learning^30, 56, 57^. We therefore subjected granule neuron-specific knockouts of RNF8 and UBC13 and control littermate mice to a delayed eye-blink conditioning response paradigm.

**Fig. 7:**
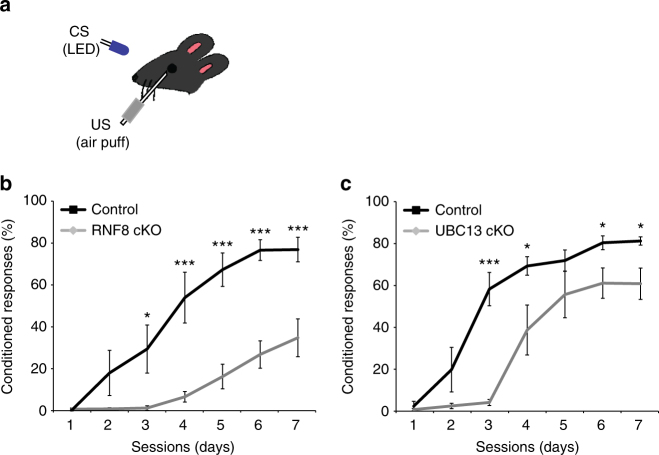
Cerebellar-dependent learning is impaired in RNF8 or UBC13 conditional knockout mice. **a** Schematic of delayed eye-blink conditioning assay depicting a mouse trained with a conditioned stimulus (CS: blue LED) paired with an eyeblink-eliciting unconditioned stimulus (US: periocular air puff). Depletion of RNF8 (**b)** or UBC13 (**c**) impaired performance on the eye-blink conditioning task (***p < 0.005, *p < 0.05, ANOVA followed by Fisher’s PLSD post hoc test)

Eye-blink conditioning allows for high-precision measurements and is sensitive to genetic perturbations in cerebellar signaling^30, 58–60^. Eye-blink responses were characterized using blue LED light pulses as the conditioned stimulus (CS) and corneal air puffs as the unconditioned stimulus (US) (Fig. 7a). After 7 consecutive sessions of conditioning (1 session per day; 150ms CS–US interval; 100 trials/session), control mice demonstrated acquisition of the conditioned response (CR). In contrast, RNF8 conditional knockout mice showed a profound deficit in learning compared to control littermates (day 1: control = 0.84% ± 0.45, RNF8 cKO = 1.29% ± 0.54, *p* = 0.516; day2: control = 18.51% ± 10.75, RNF8 cKO = 1.42% ± 0.62, *p* = 0.086; day3: control = 29.88% ± 11.45, RNF8 cKO = 1.79% ± 1.08, *p* = 0.014; day4: control = 54.30% ± 12.08, RNF8 cKO = 7.16% ± 2.44, *p* = 0.001; day5: control = 67.61% ± 7.92, RNF8 cKO = 16.79% ± 5.86, *p* = 9.75E−05; day6: control = 76.9% ± 4.92, RNF8 cKO = 27.28% ± 6.5, *p* = 3.28E−05; day7: control = 77.18% ± 5.81, RNF8 cKO = 35.22% ± 8.96, *p* = 0.0015; *n* = 7 mice; mean ± s.e.m; ANOVA followed by Fisher’s PLSD post hoc test) (Fig. 7b). These results suggest that RNF8 plays a critical role in delayed eye-blink conditioning.

We also characterized the effect of conditional knockout of RNF8 in the accelerating rotarod test, in which the latency to fall from an accelerating rotarod increases over several days. We found a modest difference in the ability of RNF8 conditional knockout and control littermate animals to learn in the accelerating rotarod test (control and RNF8 cKO, *n* = 14 mice) (Supplementary Fig. 10a and Supplementary Table 3). In other analyses, little or no difference was evident in the open-field test (control and RNF8 cKO, *n* = 10 mice) (Supplementary Fig. 10b). Together, our results suggest that RNF8 may play a key role in specific forms of cerebellar-dependent learning.

We next asked whether UBC13 plays a role in cerebellar-dependent learning. We found that UBC13 conditional knockout mice demonstrated impairment in learning compared with control littermates in the delayed eye-blink conditioning paradigm (day1: control = 2.69% ± 2.33, UBC13 cKO = 1.06% ± 0.55, *p* = 0.48; day2: control = 20.11% ± 10.62, UBC13 cKO = 2.76% ± 1.27, *p* = 0.11; day3: control = 58.56% ± 7.94, UBC13 cKO = 4.43% ± 1.44, *p* = 5.41E−0.5; day4: control = 69.57% ± 4.42, UBC13 cKO = 39.02% ± 1.19, *p* = 0.029; day5: control = 72.17% ± 5.02, UBC13 cKO = 55.91% ± 11.10, *p* = 0.21; day6: control = 80.66% ± 3.31, UBC13 cKO = 61.37% ± 7.24, *p* = 0.042; day 7: control = 81.5% ± 1.98, UBC13 cKO = 61.09% ± 7.46, *p* = 0.036; *n* = 9 mice; mean ± s.e.m; ANOVA followed by Fisher’s PLSD post hoc test) (Fig. 7c). These results suggest that like RNF8, UBC13 is required for delayed eye-blink conditioning. Taken together, our data suggest that RNF8/UBC13 signaling in granule neurons plays a crucial role in cerebellar-dependent motor learning.

## Discussion

In this study, we have discovered a novel function for RNF8/UBC13 ubiquitin signaling in the establishment of neuronal connectivity in the mammalian brain. Inhibition of RNF8 by knockdown and conditional knockout in granule neurons of the mouse cerebellum robustly increases the number of parallel fiber presynaptic boutons and functional parallel fiber/Purkinje cell synapses in vivo. In mechanistic studies, we have found that the ubiquitin ligase RNF8 operates in the cytoplasm in neurons in concert with the E2 enzyme UBC13 to suppress synapse formation in vivo. Interaction proteomics analyses reveal that neuronal RNF8 interacts with the HECT domain protein HERC2 and scaffold protein NEURL4, and their interaction plays a critical role in the ability of RNF8 to suppress synapse differentiation in vivo. Furthermore, behavior analyses showed conditional knockout of RNF8 or UBC13 robustly impairs cerebellar-dependent learning. Collectively, our findings define RNF8 and UBC13 as components of a novel cytoplasmic ubiquitin-signaling mechanism in neurons that suppresses formation of functional synapses in the cerebellum.

Although ubiquitin ligases such as Cdc20-APC have been shown to promote presynaptic development in the mammalian brain^37^, our findings define RNF8 as the first ubiquitin ligase that actively suppresses presynaptic differentiation in the mammalian brain. What is the biological significance of suppression of synapse formation in the developing brain? One possibility is that such a mechanism might be required to prevent premature synaptogenesis in newly developing neurons prior to their integration into neural circuits. Alternatively, suppression of synaptogenesis by ubiquitin signaling might provide the means to fine-tune neuronal connectivity once synapses are formed.

Whether RNF8 function in suppression of synapse formation is relevant to the pathogenesis of autism spectrum disorder is an interesting question for future studies. We have found that the inhibition of another autism-liked ubiquitin ligase, HERC2, induces an increase in presynaptic boutons, and inhibition of the ubiquitin ligase UBE3A in *Drosophila* has been shown to increase the number of presynaptic boutons at neuromuscular junctions^18^. Taken together, our findings suggest the exciting possibility that deregulation of presynaptic bouton differentiation may represent a common underlying pathogenic mechanism in autism.

Our study reveals fundamental differences as well as parallels between RNF8 functions in post-mitotic neurons and the role of RNF8 in DNA damage signaling in dividing cells. Whereas RNF8 operates in the nucleus in DNA damage signaling^20, 25^, we have found RNF8 resides and operates in the cytoplasm in post-mitotic neurons to suppress synapse differentiation in the brain. Although RNF8 may act via UBC13 and UBCH8 to promote K63-linked and K48-linked ubiquitination in DNA damage signaling in proliferating cells^21, 24, 47, 48^, RNF8 acts with UBC13 but not UBCH8 to suppress synapse differentiation in post-mitotic neurons. On the other hand, several components of the DNA damage signaling cascade including RNF8, UBC13, and HERC2 play crucial roles in the regulation of synapse formation in the brain, raising the intriguing possibility that the RNF8/UBC13 signaling cascade may operate as a cassette that has evolved to perform distinct functions in the nucleus in dividing cells and in the cytoplasm in post-mitotic cells. In both cases, owing to its interaction with RNF8, HERC2 may facilitate the assembly of the RNF8/UBC13 complex and thereby promote its activity. However, the association of NEURL4 with HERC2 in the control of synapse differentiation distinguishes such potential regulation from DNA damage signaling.

A key difference between RNF8/UBC13 signaling in the control of synapse differentiation and DNA damage signaling lies presumably in downstream mechanisms. In proliferating cells, RNF8 and UBC13 catalyze the K63-linked ubiquitination of histone H1, which triggers DNA damage response^23^. Because RNF8 operates in the cytoplasm to suppress synapse differentiation, neuronal substrates of RNF8/UBC13 might be adhesion molecules, synaptic vesicle proteins, active zone components, cytoskeletal, or presynaptic terminal mitochondria proteins with key roles in presynaptic differentiation^61, 62^. The identification of substrates of neuronal RNF8/UBC13 ubiquitin signaling represents an important question for future studies.

Suppression of synapse formation by RNF8/UBC13 signaling bears functional consequences on neurotransmission as demonstrated by electrophysiological analyses of acute cerebellar slices from RNF8 conditional knockout and UBC13 conditional knockout mice. The number of climbing fiber synapses may be homeostatically regulated relative to parallel fiber/Purkinje cell synapses^63–65^. In future studies, it will be important to determine the effect of increased number of parallel fiber/Purkinje cells synapses on the number and strength of climbing fiber/Purkinje cell synapses.

At the organism level, RNF8 or UBC13 conditional knockout mice demonstrate defects in cerebellar-dependent procedural memory, suggesting that increased number of parallel fiber/Purkinje cell synapses and potential consequent alterations of synaptic plasticity may trigger deficits in cerebellar-dependent behavior^66^. Whether inhibition of RNF8/UBC13 pathway in granule neurons impacts plasticity at parallel fiber/Purkinje cells synapses including long term depression and long term potentiation will require future studies. The coding mechanisms used in the cerebellum for learning and expressing a particular form of motor learning, such as the delayed eye-blink conditioning response, accelerated rotarod test, or adaptation of the vestibular ocular reflex (VOR), require distinct cerebellar modules^67^. It will be also important to determine if the morphological changes in synapse differentiation in RNF8/UBC13 knockout mice induce deficits in other forms of cerebellar learning such as VOR adaptation. Finally, it will be interesting to investigate whether increasing the number of synapses upon inhibition of RNF8/UBC13 signaling might serve a beneficial effect in pathological circumstances in which synapse number is reduced.

## Methods

### Plasmids

RNF8 was cloned from Sprague Dawley rat cDNA and inserted into the pEGFP-N1 or pEGFP-C1 vector (Clontech), and subcloned into the pCAG vector.

Silent rescue and point mutations of RNF8 were introduced by site-directed mutagenesis and confirmed by sequencing. A NES or NLS, respectively, was appended to an RNAi-resistant rescue form of RNF8 or GFP-RNF8 and subsequently inserted into pcDNA3. NEURL4 was cloned from Sprague Dawley rat cDNA and inserted into the pEGFP-C1 or pcDNA3-FLAG vector. A C-terminal fragment of HERC2 (HERC2 P6, 4294–4779aa) was cloned from rat cDNA and inserted into a pcDNA3-HA vector.

RNAi plasmids were designed as described^40^: HERC2, 5′-ctaggagatggaacaacaaac-3′; NEURL4, 5′-ttgacaagatggtggacaaac-3′; PARK2, 5′-actccctgattaaagagctcc-3′; RFWD2, 5′-ctacaaggatgtctcgtatct-3′; RNF8, 5′-cgctctaatggaagaactagc-3′; RNF8.2, 5′-cagagaagttacacggcaaat-3′; UBC13, 5′-cgcaggatcatcaaggaaacc-3′; UBCH8, 5′-tggaagaacctgtgcgtttgg-3′; UBE3A, 5′-ctacctaactgaagagaaagt-3′.

### Animals

Rodents were purchased or maintained under pathogen-free conditions. All animal experiments were done according to protocols approved by the Animal Studies Committee of Washington University School of Medicine and in accordance with the National Institute of Health guidelines. *UBC13* ^*loxP*/*loxP*^ and *GABAα6-Cre* have been described^49, 68^.

To generate *RNF8* ^*loxP*/*loxP*^ mice, a loxP/FRT Neo cassette was placed flanking exons 6 and 7 of the *RNF8* gene, as shown in Supplementary Fig. 2a. The targeting construct was transfected by electroporation into BA1 (C57BL/6 × 129/SvEv) hybrid embryonic stem (ES) cells. Targeted BA1 hybrid ES cells were microinjected into C57BL/6 blastocysts. Resulting chimeras with a high percentage agouti coat color were mated to C57BL/6 FLP mice to remove the Neo cassette. The gene targeting and generation of *RNF8* ^*loxP*/*loxP*^ mice were performed by Ingenious Targeting Laboratory. The *RNF8* ^*loxP*/*loxP*^ mice were backcrossed onto C57BL/6 background. For the RNF8 conditional knockout granule neuron-specific mice (within the cerebellar cortex), *RNF8* ^*loxP*/*loxP*^ mice were mated with transgenic mice expressing Cre recombinase under the promoter of the *Math1* gene (*B6.Cg-Tg*(*Atoh1-cre*)*1Bfri*/*J*, Jackson Laboratory)^41^. Genotyping for the *RNF8 flox* allele was performed with the following PCR primers: 5′-GGTTACCACTCCATAACCATCTGTACG-3′; 5′-CAGAAGGTAGCAACAGAACACGACG-3′.

### Antibodies

Antibodies to GluR2 (75–002, NeuroMab), Bassoon (ADI-VAM-PS003-F, Enzo Life Sciences), DsRed (632496, Clonetech), 14-3-3 (sc-1657, Santa Cruz Biotechnology), Tubulin (T5326, Sigma), NeuroD1 (ab60704, Abcam), HERC2 (612366, BD Bioscience), Flag (A2220, Sigma M2), ERK1/2 (9102, Cell Signaling), Hoechst (B2883, Sigma), Cre (69050, Millipore), UBC13 (37-1100, Invitrogen), HA (MMS-101P, Covance), Parp (Cell Signaling), GFP (A6455, Invitrogen/ab3970, Abcam), goat serum (G9023, Sigma), rabbit IgG (12-370, Millipore), and mouse IgG (sc-2025, Santa Cruz) were purchased. The NEURL4 antibody was kindly provided by Brian Dynlacht (NYU Cancer Institute).

A rabbit RNF8 antibody was raised against bacterially produced glutathione-*S*-transferase fusion protein containing 100–250 aa of RNF8 and purified by affinity chromatography.

Uncropped western blot images are shown in Supplementary Fig. 11.

### Subcellular fractionation

P8 rat cerebella were dissected and homogenized 25 times using a Dounce tissue grinder with hypotonic buffer (10 mM HEPES pH7.9, 10 mM KCl, 1.5 mM MgCl_2_). The homogenate was transferred to a falcon tube and centrifuged for 10 min at 800 × *g*. The supernatant (cytoplasmic fraction) was recovered. The remaining pellet was washed with the hypotonic buffer and centrifuged at 13,600 × *g* for 10 min. The pellet was resuspended in extraction buffer (20 mM HEPES pH7.9, 20% glycerol, 250 mM NaCl, 1.5 mM MgCl_2_, 0.2 mM EDTA), homogenized 10 times and incubated at 4 °C for 20 min with gentle mixing. The nuclear fraction was separated by centrifugation at 15,000 × *g* for 10 min. The supernatant was recovered as the nuclear fraction.

### Immunoprecipitation

Immunoprecipitation was performed using P8 rat cerebellar cytoplasmic lysates. The cerebellum was homogenized with hypotonic buffer (10 mM HEPES pH7.9, 10 mM KCl, 1.5 mM MgCl_2_) to separate the cytoplasmic fraction, and nuclear lysates were prepared with an extraction buffer (20 mM HEPES pH7.9, 20% glycerol, 250 mM NaCl, 1.5 mM MgCl_2_, 0.2 mM EDTA). The RNF8 antibody, coupled to protein G beads (GE Healthcare), was incubated overnight at 4 °C with cytoplasmic fractions of cerebella lysates and washed with the following buffer (50 mM Tris-HCl pH8.0, 150 mM NaCl, 0.5% Triton X-100), and immunoprecipitates were subjected to immunoblotting analyses.

Lysates of Neuro2A cells constitutively expressing FLAG-RNF8 were prepared with lysis buffer (50 mM Tris-HCl pH8.0, 150 mM NaCl, 20% glycerol, 1% Triton X-100 and proteinase inhibitor cocktail), incubated on ice for 20 min, and incubated with FlagM2 agarose beads (Sigma) for 4 h. The beads were washed with lysis buffer and the immunoprecipitates were subjected to immunoblotting analyses.

Lysates of 293T cells were transfected with an expression plasmid encoding HA-HERC2 P6, Flag-NEURL4, GFP-RNF8 WT, or GFP-RNF8 R42A. 293T cells were homogenized with lysis buffer (50 mM Tris-HCl pH8.0, 150 mM NaCl, 1% Triton X-100, and proteinase inhibitor cocktail), incubated on ice for 20 min, and incubated with the HA antibody (3F10, Roche) and mixed with protein G beads (GE Healthcare) or Flag M2 agarose beads for 2 h. The beads were washed with lysis buffer four times, and the immunoprecipitates were subjected to immunoblotting analyses.

### Immunoprecipitation mass spectrometry

Immunoprecipitation was performed in Neuro2A cells. Neuro2A cells constitutively expressing Flag-RNF8 were generated by single colony isolation and maintained in the presence of 0.5 mg/ml of G418. Lysate was prepared with lysis buffer (50 mM Tris-HCl pH8.0, 150 mM NaCl, 1% Triton X-100, and proteinase inhibitor cocktail) and incubated on ice for 20 min. Lysates were incubated with FlagM2 agarose beads (Sigma) for 4 h. The beads were washed with wash buffer (50 mM Tris-HCl pH8.0, 150 mM NaCl, 0.1% Triton X-100), and the immunoprecipitates were eluted by 3×FLAG peptide (Sigma), followed by TCA precipitation. Proteins were trypsinized (Sequencing-Grade Trypsin, Promega) and washed (3M Empore C18 media), and tryptic peptides were loaded onto an LTQ linear ion trap mass spectrometer (ThermoFinningan). Spectra were searched against target-decoy mouse tryptic peptide databases. Com*PASS* analysis was done against dedicated Neuro2A (>60 bait-runs) IP-MS databases.

### Cerebellar granule neuron cultures

Granule neurons were prepared from the cerebellum of P6 Long Evans rat pups as described^69^. High-efficiency transfection of granule neurons (maximum efficiency 80%) was achieved using a nucleofection method with Amaxa electroporation device as described^38^.

### In vivo electroporation and immunohistochemistry

In vivo electroporation of postnatal rat pups was performed as described^35–37^. P4 Sprague Dawley rat pups or P6–P9 mouse pups were injected with the indicated plasmids, and subjected to five electric pulses of 175 mV (rat) or 135 mV (mouse) with 950 ms intervals. Electroporated pups were returned to moms and examined 4, 8, or 12 days later following immunohistochemistry analyses. Rat or mouse pups were fixed with 4% PFA and 4% sucrose and labeled with the relevant antibodies. Images were taken in a blinded manner using a NIKON eclipse TE2000 epifluorescence microscope using a digital CCD camera (DIAGNOSTIC instruments) and analyzed using the SPOT imaging software. Images of the synaptic proteins Bassoon and GluR2 at parallel fiber GFP-positive varicosities were taken using a Olympus FV1200 Confocal microscope.

### Electron microscopy

P24–P25 mice were perfused with 2% formaldehyde and 2.5% glutaraldehyde in 0.1 M sodium cacodylate buffer, pH 7.4. The cerebellum was collected, postfixed overnight, and stored in fixative solution, and embedded in 4% agar. Sections (0.9 mm) were cut sagittally, rinsed in 0.15 M cacodylate buffer 3 times for 10 min each, and subjected to a secondary fixation step for 1 h in 1% osmium tetroxide/1.5% potassium ferrocyanide in cacodylate buffer on ice. Samples were next washed in ultrapure water 3 times for 10 min each and en bloc stained for 1 h with 2% aqueous uranyl acetate. After staining was complete, samples were briefly washed in ultrapure water, dehydrated in a graded acetone series (50%, 70%, 90%, 100% × 2) for 10 min in each step, infiltrated with microwave assistance (Pelco BioWave Pro, Redding, CA) into LX112 resin, and flat embedded between two slides that had been coated with PTFE release agent (Miller-Stephenson #MS-143XD, Danbury, CT) and clamped with binder clips. Samples were cured in an oven at 60˚C for 48 h. Slides were separated and mounted on a blank stub with epoxy. In total, 70 nm thin sections were then taken, counterstained with uranyl acetate and lead citrate, and imaged on a TEM (JEOL JEM-1400 Plus, Tokyo, Japan) at 80 KeV with an AMT XR111 camera.

### Electrophysiology

Acute 300 μm sagittal and coronal slices were prepared from the cerebellum of P20–P25 RNF8 cKO and control *RNF8* ^*loxP*/*loxP*^ or P23–P26 UBC13 cKO and control *UBC13* ^*loxP*/*loxP*^ mice. Slices were cut in dissecting solution containing: 83 mM NaCl, 65 mM sucrose, 26 mM NaHCO_3_, 25 mM glucose, 6.8 mM MgCl_2_, 2.5 mM KCl, 1.25 NaH_2_PO_4_, and 0.5 CaCl_2_. Slices were incubated at 35 °C for 1 h in artificial cerebrospinal fluid (ACSF) containing 125 mM NaCl, 26 mM NaHCO_3_, 1.25 mM NaH_2_PO_4_, 2.5 mM KCl, 1 mM MgCl_2_, 2 mM CaCl_2_, and 25 mM glucose, and switched to room temperature prior to recording. Slice solutions were constantly bubbled with 95% O_2_ and 5% CO_2_. Electrophysiological signals were acquired with a Multiclamp 700B amplifier, digitized at 10 kHz with a Digidata 1440 A D-A converter, and Bessel filtered at 2 kHz.

Whole-cell patch-clamp recordings in Purkinje cells were obtained with electrodes (2–3 MΩ) filled with intracellular solution containing 130 mM Cs-methanesulfonate, 5 mM CsCl, 10 mM HEPES, 0.5 mM EGTA, 2 mM MgCl_2_, 2 mM Na_2_-ATP, and 0.5 mM Na_2_-GTP for mEPSC recordings, or with intracellular solution containing 125 mM K-gluconate, 15 mM KCl, 10 mM HEPES, 2 mM Mg-ATP, 0.3 mM Na_2_-GTP, 10 mM Na_2_-phosphocreatine, 0.2 mM EGTA, and 10 mM Qx-314 for evoked EPSC recordings. Purkinje neurons were voltage-clamped at −70 mV. For mEPSC recordings, 1 μM tetrodotoxin and 20 μM picrotoxin was added to ACSF. The parameters of mEPSC analyses in the MiniAnalysis software used were: threshold: 6 pA; period to search a local maximum (μs): 10,000; time before a peak for baseline (μs): 8000; period to search a decay time (μs): 20,000; fraction of peak to find decay time: 0.37; period to average a baseline (μs): 1000; area threshold: 15; number of points to average for peak: 1. For evoked EPSC recordings, 20 μM picrotoxin was added to ACSF. mEPSC traces were additionally low-pass filtered at 1 kHz. For evoked EPSC and presynaptic volley recordings, the molecular layer was stimulated with a bipolar concentric electrode using brief (0.1 ms) current pulses. Evoked EPSCs were measured using the whole-cell recording electrodes as described above. Extracellular presynaptic volley recordings using 1 MΩ electrodes filled with 3 M NaCl were made 400 μm away from the site of stimulation^70^. The fiber volley amplitude was derived from the negative-going phase of the extracellular field potential. Whole-cell patch-clamp recordings in cerebellar granule neurons were obtained with electrodes (7–8 MΩ) filled with intracellular solution containing 125 mM K-gluconate, 15 mM KCl, 10 mM HEPES, 2 mM Mg-ATP, 0.3 mM Na_2_-GTP, 10 mM Na_2_-phosphocreatine and 0.2 mM EGTA. For electrophysiological properties of intrinsic excitability of cerebellar granule neurons, a series of depolarizing current steps (10, 20, 30, 40 pA) of 500 ms duration under current clamp were applied to the recorded cerebellar granule neuron. The evoked AP patterns were recorded and the number and amplitude of APs during the 500 ms depolarizing current injection step and the input resistance were measured. For paired-pulse facilitation at parallel fiber/Purkinje cell synapses, a paired electrical stimulation with a 50 ms inter-pulse interval was applied to the parallel fiber bundles and the evoked EPSCs were recorded in the Purkinje cells. Paired-pulse facilitation was measured by calculating the ratio of the second EPSC amplitude to the first EPSC amplitude.

### qRT-PCR

Reverse transcription reactions were performed with Superscript III (Invitrogen) according to manufacturer’s protocol. Real-time PCR reactions were performed using Lightcycler 480 SYBR Green I Master (Roche). The following primers were used: Gapdh forward, 5′-tgctggtgctgagtatgtcg-3′; Gapdh reverse, 5′-gcatgtcagatccacaacgg-3′; HERC2 forward, 5′-atctctgcagtgagctcttgcag-3′; HERC2 reverse, 5′-actccagtagaatggccaaagcc-3′; NEURL4 forward, 5′-ccatgaccaatttacgctctggg-3′; NEURL4 reverse, 5′-gatcaacgacagcatagacaccc-3′; PARK2 forward, 5′-aaggagctgcagaatcacctgac-3′; PARK2 reverse, 5′-ctgcttctgagtcactcttgctg-3′; RFWD2 forward, 5′-aggagatgagtggcttgtactcc-3′; RFWD2 reverse, 5′-tgtttctttgtctgagacggccc-3′; RNF8 mouse C-term forward, 5′-attgaggctgtcaccctgaactg-3′; RNF8 mouse C-term reverse, 5′-gtccttattagggaacggagagc; RNF8 mouse N-term, RNF8 rat forward, 5′-agaatcctgagggacaatggacg-3′; RNF8 rat reverse, 5′-tccgttgtgtggtcattcttggg-3′; UBC13 mouse forward, 5′-gaactcgggatctgacaagatgg-3′; UBC13 mouse reverse, 5′-gctgccattgggtattcttctgg-3′; UBCH8 forward, 5′-gtggctaaagagctggacgatct-3′; UBCH8 reverse, 5′-ggtggtgaatctcaaagtgggag-3′; UBE3A forward, 5′-ctatgcaaatgtagtgggagggg-3′; UBE3A reverse, 5′-gcactggttcagagctttcggaa-3′.

### Delayed eye-blink conditioning assay

The delayed eye-blink conditioning assay was performed as described with modifications^60^. UBC13 conditional knockout, RNF8 conditional knockout, or littermate control mice at 8-9 weeks of age were used. Mice anesthetized with ketamine/xylazine (100 mg/kg; 10 mg/kg) were implanted with a head plate placed over the skull bregma and secured with screws using Metabond cement (Parkell). Five days following recovery from surgery, head-fixed mice were habituated on a cylindrical treadmill over two 2 h sessions. After training, mice were tested with the head-fixed eye-blink conditioning apparatus. Mice learned to blink in response to an initially neutral CS (blue LED) that is paired with an eyeblink-eliciting US 20 psi periocular air puff through a flattened 25 gauge needle; CS–US interstimulus interval, 150 msec). 100 trials of CS and US pairing were performed for each day, and learned eyelid CR was tracked over 7 consecutive days using a high-speed monochrome camera (Allied Vison). The eyelid position was calculated on each frame by the “pixel area” method described previously^60^, and quantified in units of fraction eyelid closure, ranging from 0 (fully open) to 1 (fully closed). Eyelid closure >0.1 during the ISI period was defined as a CR. The percentage of trials during each session containing CRs was used as the index of motor learning.

### Accelerating rotarod behavior assay

The accelerating rotarod assay was performed using RNF8 conditional knockout or control littermate mice at 8–9 weeks of age. On the first day, mice were habituated on the rotarod apparatus (IITC RotaRod) rotating at a constant 5 rotations per minute (rpm). Mice were then tested on the rotarod, which was accelerating from 5 to 40 rpm over a period of 3 min and the latency to which each mouse fell from the rod onto the sensing platform below was recorded automatically. Each daily session consisted of three trials with a 10 min intertrial interval, and each mouse was tested for five sessions over sequential days.

### Open-field assay

The open-field assay was performed using 6–8-week-old littermate RNF8 conditional knockout or control littermate mice. Mice were placed in the center of the open-field arena (45 × 23 cm) and allowed to move freely for 15 minutes. The position of mice was tracked by a video camera and analyzed using a custom-written MATLAB script.

### Statistics

Statistical analyses were done using GraphPad Prism 6.0 software. Bar graphs are presented as the mean ± SEM. For experiments in which only two groups were analyzed, the *t* test was used. Pairwise comparisons within multiple groups were done by analysis of variance (ANOVA) followed by the Fisher’s PLSD post hoc test. Kolmogorov–Smirnov test was used for analyses of the cumulative distribution of EPSC amplitude and frequency.

### Data availability

The data that support the findings of this study are available from the corresponding author upon reasonable request.

## Electronic supplementary material

Supplementary Information

## References

[1] Yamada T, Yang Y, Bonni A (2013). Spatial organization of ubiquitin ligase pathways orchestrates neuronal connectivity. Trends Neurosci..

[2] Yi JJ, Ehlers MD (2007). Emerging roles for ubiquitin and protein degradation in neuronal function. Pharmacol. Rev..

[3] Hershko A, Ciechanover A (1998). The ubiquitin system. Annu. Rev. Biochem..

[4] Komander D (2009). The emerging complexity of protein ubiquitination. Biochem. Soc. Trans..

[5] Lehman NL (2009). The ubiquitin proteasome system in neuropathology. Acta Neuropathol..

[6] Jiang YH, Beaudet AL (2004). Human disorders of ubiquitination and proteasomal degradation. Curr. Opin. Pediatr..

[7] Clayton-Smith J, Laan L (2003). Angelman syndrome: a review of the clinical and genetic aspects. J. Med. Genet..

[8] Kishino T, Lalande M, Wagstaff J (1997). UBE3A/E6-AP mutations cause Angelman syndrome. Nat. Genet..

[9] Matsuura T (1997). De novo truncating mutations in E6-AP ubiquitin-protein ligase gene (UBE3A) in Angelman syndrome. Nat. Genet..

[10] Bruinsma CF (2015). Dissociation of locomotor and cerebellar deficits in a murine Angelman syndrome model. J. Clin. Invest..

[11] Glessner JT (2009). Autism genome-wide copy number variation reveals ubiquitin and neuronal genes. Nature.

[12] Scheuerle A, Wilson K (2011). PARK2 copy number aberrations in two children presenting with autism spectrum disorder: further support of an association and possible evidence for a new microdeletion/microduplication syndrome. Am. J. Med. Genet. B. Neuropsychiatr. Genet..

[13] Yin CL (2016). Genome-wide analysis of copy number variations identifies PARK2 as a candidate gene for autism spectrum disorder. Mol. Autism.

[14] Puffenberger EG (2012). A homozygous missense mutation in HERC2 associated with global developmental delay and autism spectrum disorder. Hum. Mutat..

[15] Morrow EM (2008). Identifying autism loci and genes by tracing recent shared ancestry. Science.

[16] Buhlman LM (2016). Parkin loss-of-function pathology: premature neuronal senescence induced by high levels of reactive oxygen species?. Mech. Ageing Dev..

[17] Geisler S (2010). PINK1/Parkin-mediated mitophagy is dependent on VDAC1 and p62/SQSTM1. Nat. Cell Biol..

[18] Li W (2016). Angelman syndrome protein Ube3a regulates synaptic growth and endocytosis by inhibiting BMP signaling in drosophila. PLoS Genet..

[19] Mabb AM, Judson MC, Zylka MJ, Philpot BD (2011). Angelman syndrome: insights into genomic imprinting and neurodevelopmental phenotypes. Trends Neurosci..

[20] Mailand N (2007). RNF8 ubiquitylates histones at DNA double-strand breaks and promotes assembly of repair proteins. Cell.

[21] Bekker-Jensen S (2010). HERC2 coordinates ubiquitin-dependent assembly of DNA repair factors on damaged chromosomes. Nat. Cell Biol..

[22] Huen MS (2007). RNF8 transduces the DNA-damage signal via histone ubiquitylation and checkpoint protein assembly. Cell.

[23] Thorslund T (2015). Histone H1 couples initiation and amplification of ubiquitin signalling after DNA damage. Nature.

[24] Lok GT (2012). Differential regulation of RNF8-mediated Lys48- and Lys63-based poly-ubiquitylation. Nucleic Acids Res..

[25] Feng L, Chen J (2012). The E3 ligase RNF8 regulates KU80 removal and NHEJ repair. Nat. Struct. Mol. Biol..

[26] Hatten ME, Heintz N (1995). Mechanisms of neural patterning and specification in the developing cerebellum. Annu. Rev. Neurosci..

[27] de la Torre-Ubieta L, Bonni A (2011). Transcriptional regulation of neuronal polarity and morphogenesis in the mammalian brain. Neuron.

[28] Puram SV, Bonni A (2013). Cell-intrinsic drivers of dendrite morphogenesis. Development.

[29] Tsai PT (2012). Autistic-like behaviour and cerebellar dysfunction in Purkinje cell Tsc1 mutant mice. Nature.

[30] Kloth AD (2015). Cerebellar associative sensory learning defects in five mouse autism models. Elife.

[31] Wang SS, Kloth AD, Badura A (2014). The cerebellum, sensitive periods, and autism. Neuron.

[32] Maximo JO, Cadena EJ, Kana RK (2014). The implications of brain connectivity in the neuropsychology of autism. Neuropsychol. Rev..

[33] Geschwind DH, Levitt P (2007). Autism spectrum disorders: developmental disconnection syndromes. Curr. Opin. Neurobiol..

[34] Bourgeron T (2015). From the genetic architecture to synaptic plasticity in autism spectrum disorder. Nat. Rev. Neurosci..

[35] Konishi Y, Stegmuller J, Matsuda T, Bonni S, Bonni A (2004). Cdh1-APC controls axonal growth and patterning in the mammalian brain. Science.

[36] Shalizi A (2006). A calcium-regulated MEF2 sumoylation switch controls postsynaptic differentiation. Science.

[37] Yang Y (2009). A Cdc20-APC ubiquitin signaling pathway regulates presynaptic differentiation. Science.

[38] Yamada T (2013). Sumoylated MEF2A coordinately eliminates orphan presynaptic sites and promotes maturation of presynaptic boutons. J. Neurosci..

[39] Gaudilliere B, Konishi Y, de la Iglesia N, Yao G, Bonni A (2004). A CaMKII-NeuroD signaling pathway specifies dendritic morphogenesis. Neuron..

[40] Gaudilliere B, Shi Y, Bonni A (2002). RNA interference reveals a requirement for myocyte enhancer factor 2A in activity-dependent neuronal survival. J. Biol. Chem..

[41] Schuller U (2007). Forkhead transcription factor FoxM1 regulates mitotic entry and prevents spindle defects in cerebellar granule neuron precursors. Mol. Cell Biol..

[42] Lu LY (2010). RNF8-dependent histone modifications regulate nucleosome removal during spermatogenesis. Dev. Cell.

[43] Bandeira F, Lent R, Herculano-Houzel S (2009). Changing numbers of neuronal and non-neuronal cells underlie postnatal brain growth in the rat. Proc. Natl Acad. Sci. USA.

[44] Kim AH (2009). A centrosomal Cdc20-APC pathway controls dendrite morphogenesis in postmitotic neurons. Cell.

[45] Stegmuller J (2006). Cell-intrinsic regulation of axonal morphogenesis by the Cdh1-APC target SnoN. Neuron.

[46] Litterman N (2011). An OBSL1-Cul7Fbxw8 ubiquitin ligase signaling mechanism regulates Golgi morphology and dendrite patterning. PLoS Biol..

[47] Ito K (2001). N-Terminally extended human ubiquitin-conjugating enzymes (E2s) mediate the ubiquitination of RING-finger proteins, ARA54 and RNF8. Eur. J. Biochem..

[48] Plans V (2006). The RING finger protein RNF8 recruits UBC13 for lysine 63-based self polyubiquitylation. J. Cell Biochem..

[49] Funfschilling U, Reichardt LF (2002). Cre-mediated recombination in rhombic lip derivatives. Genesis.

[50] Sowa ME, Bennett EJ, Gygi SP, Harper JW (2009). Defining the human deubiquitinating enzyme interaction landscape. Cell.

[51] Zhang C (2013). The X-linked intellectual disability protein PHF6 associates with the PAF1 complex and regulates neuronal migration in the mammalian brain. Neuron.

[52] Mejia LA (2013). A novel Hap1-Tsc1 interaction regulates neuronal mTORC1 signaling and morphogenesis in the brain. J. Neurosci..

[53] Al-Hakim AK, Bashkurov M, Gingras AC, Durocher D, Pelletier L (2012). Interaction proteomics identify NEURL4 and the HECT E3 ligase HERC2 as novel modulators of centrosome architecture. Mol. Cell Proteomics.

[54] Li J (2012). Neurl4, a novel daughter centriole protein, prevents formation of ectopic microtubule organizing centres. EMBO Rep..

[55] Martinez-Noel G (2012). Identification and proteomic analysis of distinct UBE3A/E6AP protein complexes. Mol. Cell Biol..

[56] Yang Y (2016). Chromatin remodeling inactivates activity genes and regulates neural coding. Science.

[57] Galliano E (2013). Silencing the majority of cerebellar granule cells uncovers their essential role in motor learning and consolidation. Cell Rep..

[58] Rinaldo L, Hansel C (2010). Ataxias and cerebellar dysfunction: involvement of synaptic plasticity deficits?. Funct. Neurol..

[59] McCormick DA, Thompson RF (1984). Cerebellum: essential involvement in the classically conditioned eyelid response. Science.

[60] Heiney SA, Wohl MP, Chettih SN, Ruffolo LI, Medina JF (2014). Cerebellar-dependent expression of motor learning during eyeblink conditioning in head-fixed mice. J. Neurosci..

[61] Jin Y, Garner CC (2008). Molecular mechanisms of presynaptic differentiation. Annu. Rev. Cell Dev. Biol..

[62] Chia PH, Li P, Shen K (2013). Cell biology in neuroscience: cellular and molecular mechanisms underlying presynapse formation. J. Cell Biol..

[63] Hirai H (2005). Cbln1 is essential for synaptic integrity and plasticity in the cerebellum. Nat. Neurosci..

[64] Watanabe M, Kano M (2011). Climbing fiber synapse elimination in cerebellar Purkinje cells. Eur. J. Neurosci..

[65] Hashimoto K (2009). Influence of parallel fiber-Purkinje cell synapse formation on postnatal development of climbing fiber-Purkinje cell synapses in the cerebellum. Neuroscience.

[66] Le Guen MC, De Zeeuw CI (2010). Presynaptic plasticity at cerebellar parallel fiber terminals. Funct. Neurol..

[67] De Zeeuw CI, Ten Brinke MM (2015). Motor learning and the cerebellum. Cold Spring Harb. Perspect. Biol..

[68] Yamamoto M (2006). Key function for the Ubc13 E2 ubiquitin-conjugating enzyme in immune receptor signaling. Nat. Immunol..

[69] Bilimoria PM, Bonni A (2008). Cultures of cerebellar granule neurons. CSH Protoc..

[70] Sabatini BL, Regehr WG (1997). Control of neurotransmitter release by presynaptic waveform at the granule cell to Purkinje cell synapse. J. Neurosci..

