# Supplementary material for: RNF8/UBC13 ubiquitin signaling suppresses synapse formation in the mammalian brain
Source: Nat Commun. 2017 Nov 2;8:1271. doi: 10.1038/s41467-017-01333-6 (PMC5668370; doi:10.1038/s41467-017-01333-6)
Supplement: Supplementary file 1 — Supplementary Information [file 41467_2017_1333_MOESM1_ESM.pdf]

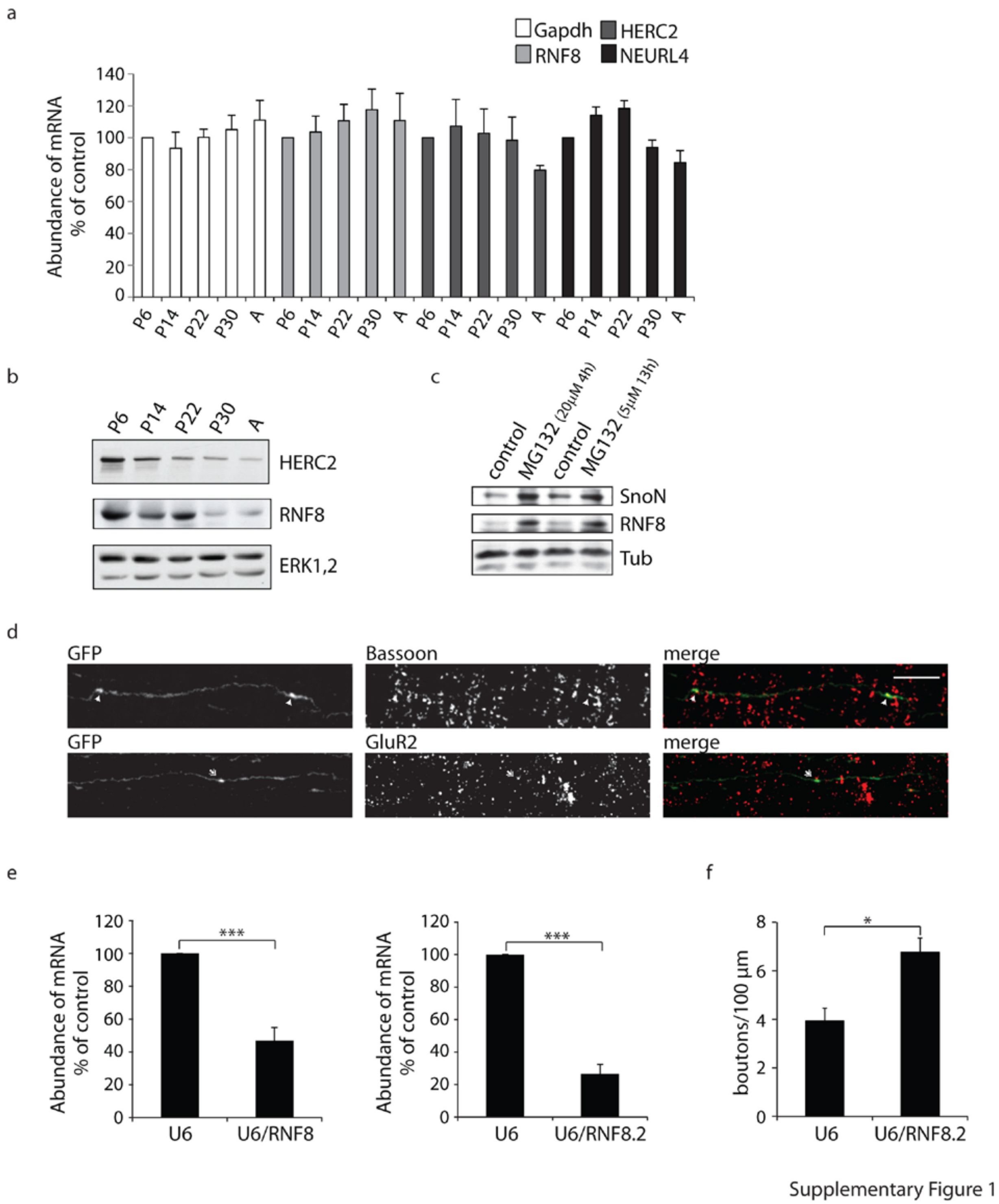

Supplementary Figure 1: Expression of RNF8, HERC2 and NEURL4 in the cerebellum and knockdown of RNF8 by RNAi

(a) Lysates of the cerebellum from rat pups at P6, P14, P22, P30 and adult (A) rats were subjected to quantitative RT-PCR (qRT-PCR) analyses using primers specific to RNF8, HERC2 and NEURL4, along with Gapdh, the latter serving as control. RNF8, HERC2 and NEURL4

mRNA were highly expressed in the cerebellum during the second and third weeks of postnatal development. (b) Lysates of the cerebellum from rats at different ages were subjected to immunoblotting analyses. RNF8 and HERC2 protein were expressed in two- and three-week old pups, though RNF8 and HERC2 protein declined into adulthood. (c) Lysates of granule neurons at DIV3-4 treated with MG132 (20μm for 4hr or 5μm for 13hrs) were immunoblotted with the RNF8, SnoN and Tubulin antibodies. RNF8 protein levels increased upon inhibition of the proteasome. (d) P4 rat pups were electroporated with the GFP expression plasmid. After 4 or 5 days, the cerebellum was removed and cerebellar sections were immunolabeled with the GFP antibody together with the Bassoon, or GluR2 antibody. Bassoon co-localized with parallel fiber GFP-positive varicosities (arrowheads), and GluR2 puncta were adjacent to GFP-positive varicosities (double arrowheads), indicating that parallel fiber GFP-labeled varicosities represent presynaptic boutons at synaptic sites. Scale bar=10μm. (e) Granule neurons were transfected with the U6/RNF8, U6/RNF8.2 or control U6 plasmid and subjected to qRT-PCR analyses using primers specific to RNF8, along with Gapdh. The levels of each mRNA normalized to Gapdh are shown relative to the control condition. RNF8 and RNF8.2 RNAi induced the knockdown of endogenous RNF8 in neurons (RNF8 RNAi, ***$p<0.005$, t test, n=3 cultures; RNF8.2 RNAi, ***$p<0.001$, t test, n=3 cultures). (f) P4 rat pups were electroporated with the RNF8.2 RNAi or control U6 plasmid together with a GFP expression plasmid and analyzed as in Fig. 1a. Knockdown of RNF8 increased the density of presynaptic parallel fiber boutons in the cerebellar cortex *in vivo* (*$p<0.05$, t test, n=3 rats).

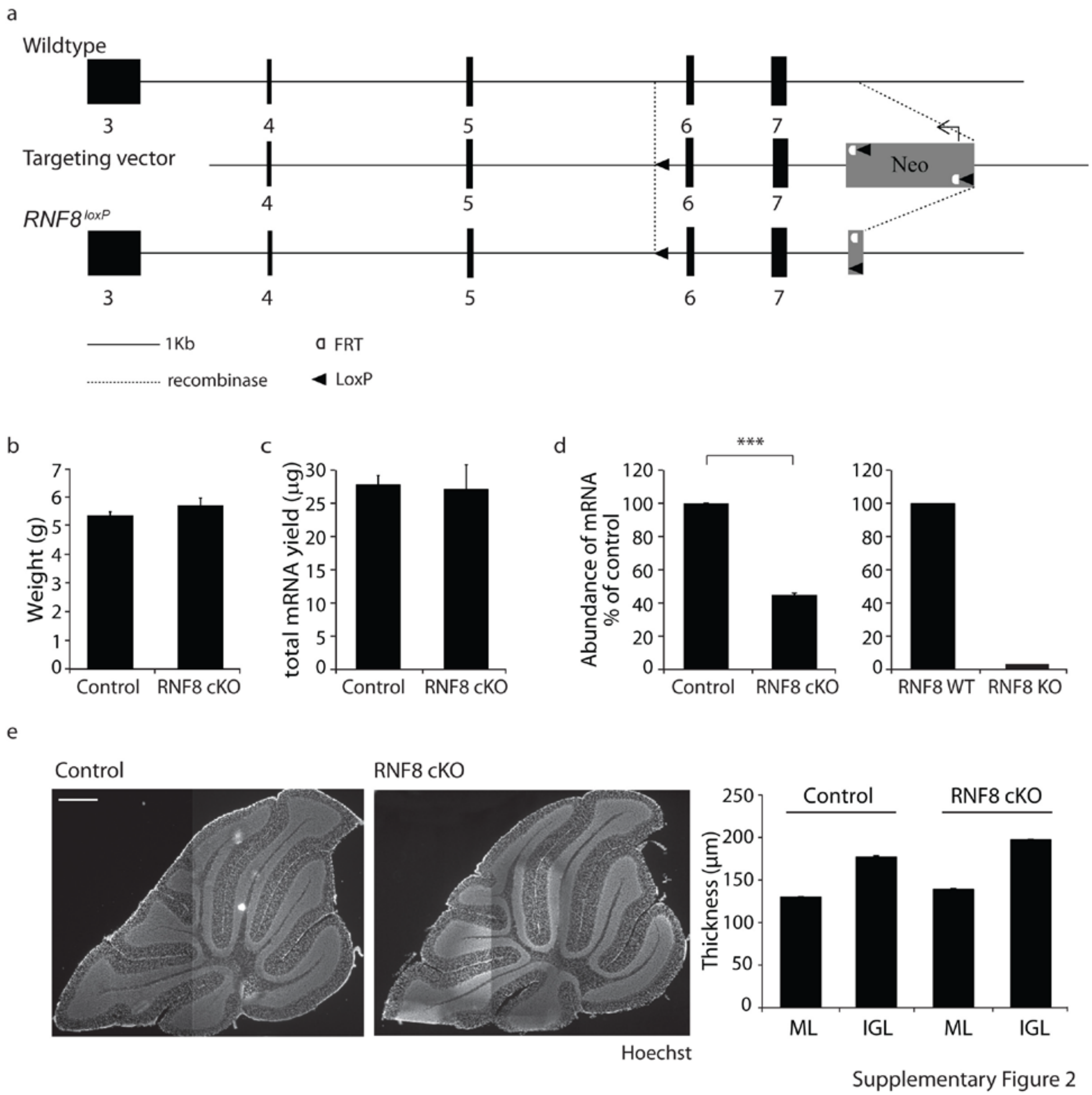

Supplementary Figure 2: Characterization of RNF8 conditional knockout mice
(a) Targeting strategy. The structure of the *RNF8* wildtype and floxed allele is shown. Exons are numbered 3 to 7 and are represented by black boxes. (b) No difference in weight was found in RNF8 conditional knockout (RNF8 cKO) compared to control $RNF8^{loxP/loxP}$ mice at P10. (c) Little or no difference was found in total mRNA levels in the cerebellum of RNF8 conditional knockout mice compared to control mice. (d) qRT-PCR analyses were performed using primers specific to RNF8, along with Gapdh, the latter serving as control, in P10 RNF8 cKO and control mice or in RNF8 full KO (RNF8 KO) and control wildtype mice. Efficiency of knockout is shown (***$p<0.001$, t test, n=3 mice). (e) P26 cerebellar sections from RNF8 cKO and control mice were subjected to immunohistochemistry using the DNA dye Hoechst 33258. Left: To visualize the full cerebellar vermis, overlapping images were assembled. Conditional knockout

of RNF8 in postmitotic granule neurons had little or no effect on the gross anatomy of the cerebellum. Scale bar=500μm. Right: little or no difference was found in the thickness of the molecular and internal granule layers in RNF8 conditional knockout and control mice.

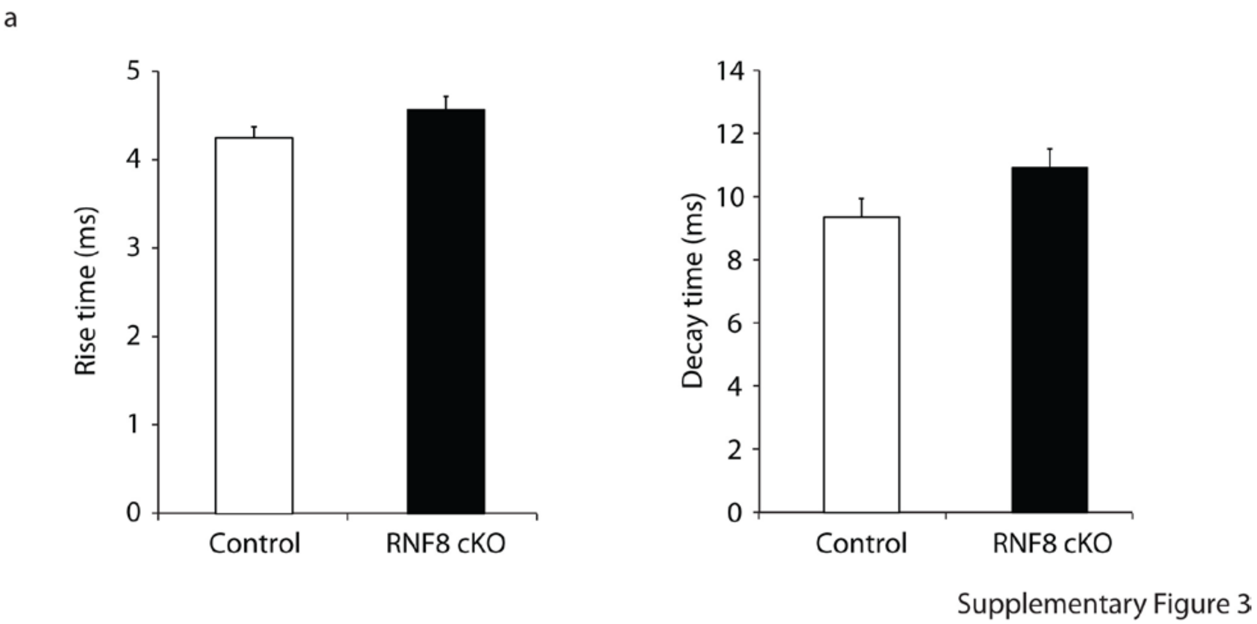

Supplementary Figure 3: Rise and decay time of mEPSCs in RNF8 conditional knockout mice

(a) Little or no difference in the rise and decay time of mEPSCs in Purkinje neurons was found in RNF8 conditional knockout and control mice (Supplementary Table 1).

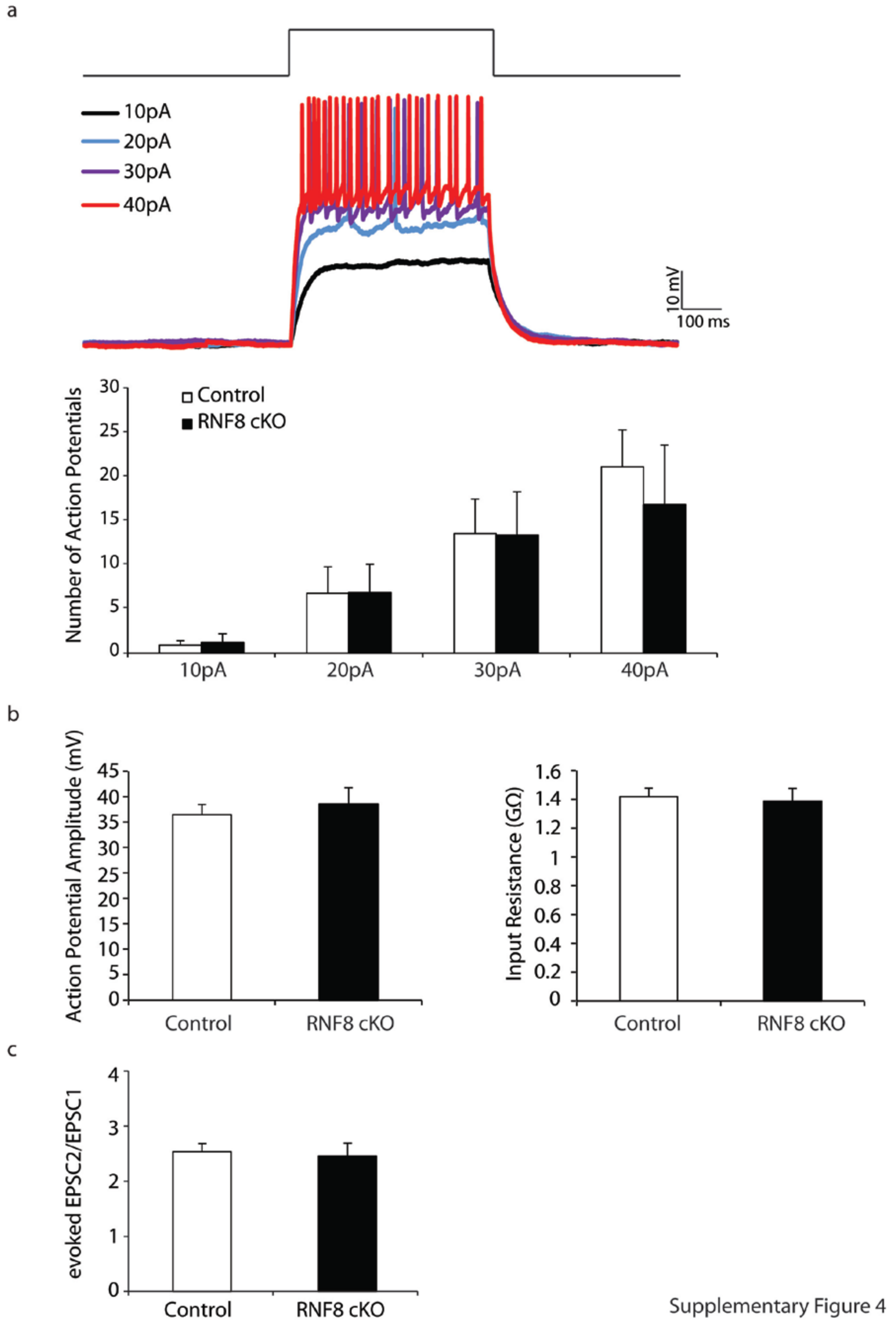

Supplementary Figure 4

Supplementary Figure 4: Intrinsic excitability and paired-pulse facilitation in RNF8 conditional knockout mice

(a-b) Electrophysiological properties of intrinsic excitability of granule neurons in the cerebellum. A series of step-depolarizing current (10, 20, 30, 40pA) of 500ms duration under current clamp were applied to the recorded granule neuron. The evoked action potential (AP) patterns were recorded and the number of APs during 500ms step-depolarizing current injection was measured (a). The APs amplitude and input resistance were also measured (b). Conditional knockout of RNF8 in granule neurons had little or no effect on the evoked AP frequency, AP amplitude, and input resistance, indicating conditional knockout of RNF8 does not affect the intrinsic excitability of granule neurons. (c) Paired-pulse facilitation at parallel fiber/Purkinje cell synapses. A paired electrical stimulation (50ms inter-pulse interval) was applied to parallel fibers, and the evoked EPSCs were recorded in Purkinje cells. Paired-pulse facilitation was measured by calculating the ratio of the amplitude of the second EPSC to the first EPSC. Conditional knockout of RNF8 in granule neurons had little or no effect on paired-pulse facilitation at parallel fiber/Purkinje cell synapses.

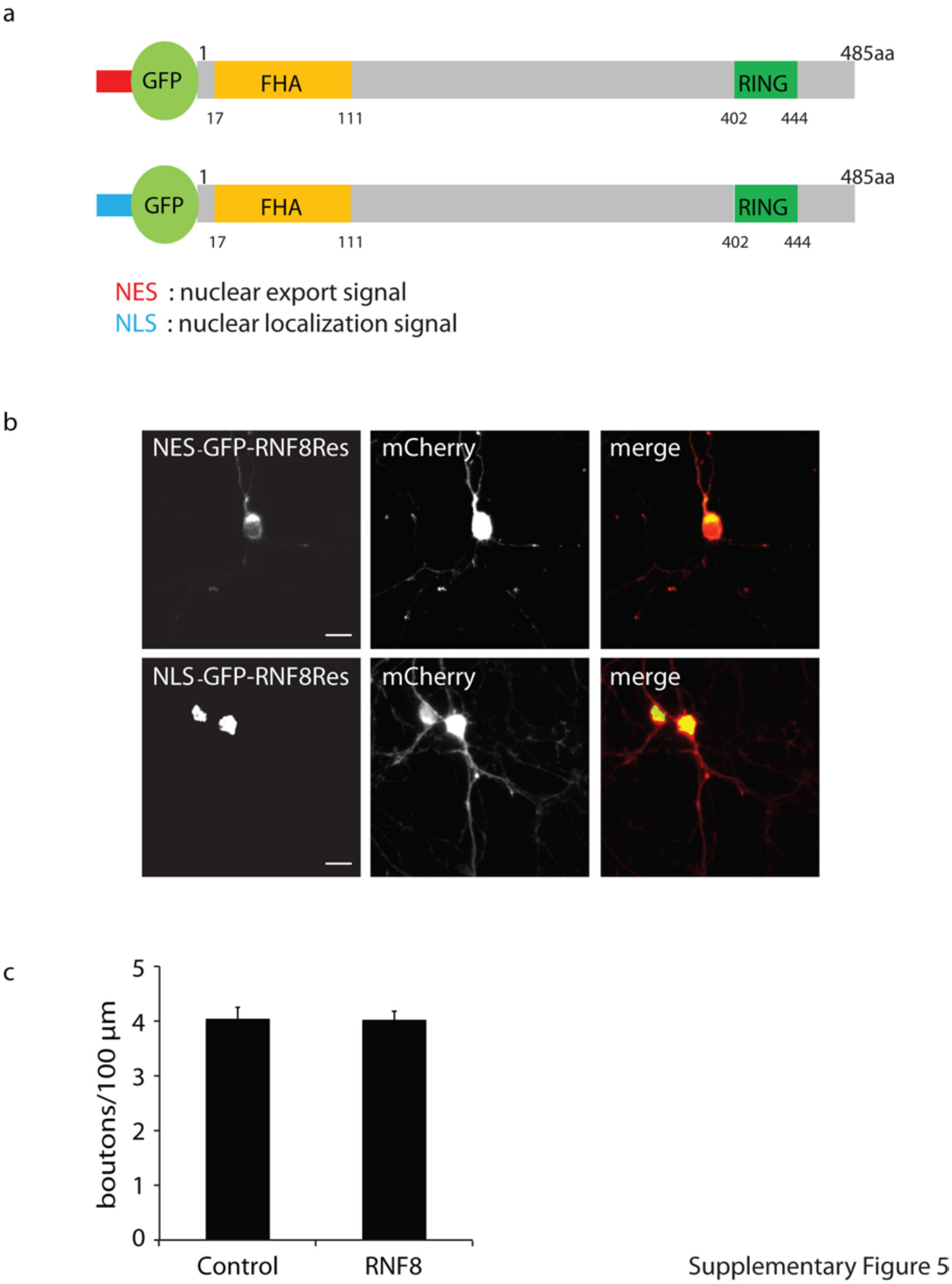

Supplementary Figure 5: Nuclear and cytoplasmic localization of RNF8
(a) Schematic representation of an expression plasmid encoding NES (nuclear export signal)-GFP-RNF8Res fusion protein or NLS (nuclear localization signal)-GFP-RNF8Res fusion

protein. (b) Primary granule neurons prepared from P6 rat pups were transfected with an expression plasmid encoding NES-GFP-RNF8Res fusion protein or NLS-GFP-RNF8Res fusion protein together with an mCherry expression plasmid at 2 days *in vitro* (DIV2). At DIV4, granule neurons were subjected to immunocytochemistry using the GFP and DsRed (mCherry) antibodies. NES-GFP-RNF8Res and NLS-GFP-RNF8Res were localized predominantly in the cytoplasm and nucleus, respectively. Scale bars=10μm. (c) Exogenous expression of RNF8 had little or no effect on the number of parallel fiber boutons *in vivo*.

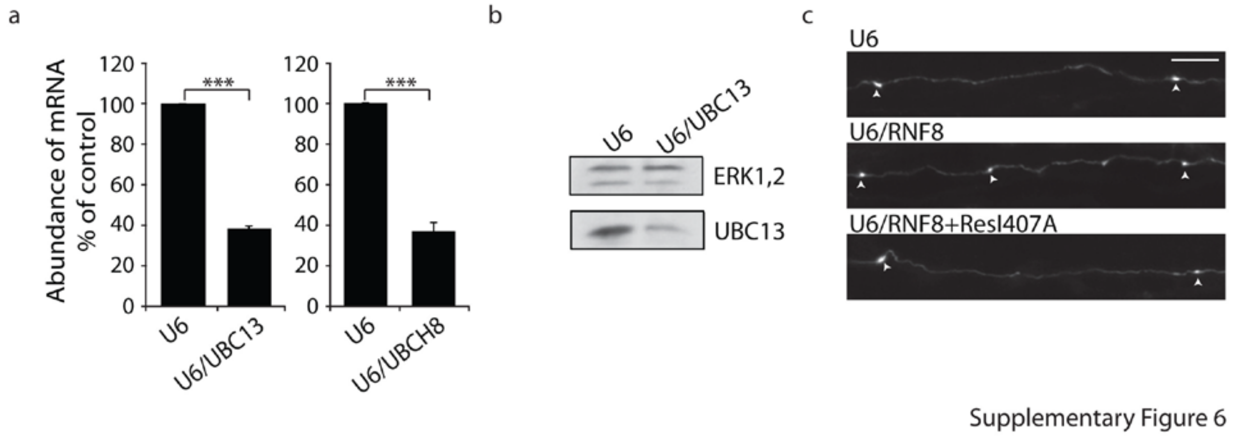

Supplementary Figure 6: Identification of RNF8/E2 pathway

(a) Granule neurons were transfected with the U6/UBC13, U6/UBCH8 or control U6 plasmid and subjected to qRT-PCR analyses using primers specific to UBC13 or UBCH8, along with Gapdh. The levels of each mRNA normalized to Gapdh are shown relative to the control condition. UBC13 RNAi and UBCH8 RNAi induced the knockdown of endogenous UBC13 (***$p<0.001$, t test, n=3 cultures) and endogenous UBCH8 (***$p<0.001$, t test, n=5 cultures), respectively, in neurons. (b) Lysates of granule neurons electroporated with the U6/UBC13 or control U6 plasmid were immunoblotted with the UBC13 and ERK antibodies, the latter to serve as a loading control. UBC13 RNAi reduced the abundance of endogenous UBC13 protein in neurons. (c) Images of axons from rat pups electroporated at P4 with the control U6 plasmid or RNF8 RNAi together with a RNF8Res mutant in which isoleucine 407 was replaced with alanine (RNF8ResI407A) or the control vector and with the GFP expression plasmid. Scale bar=10μm.

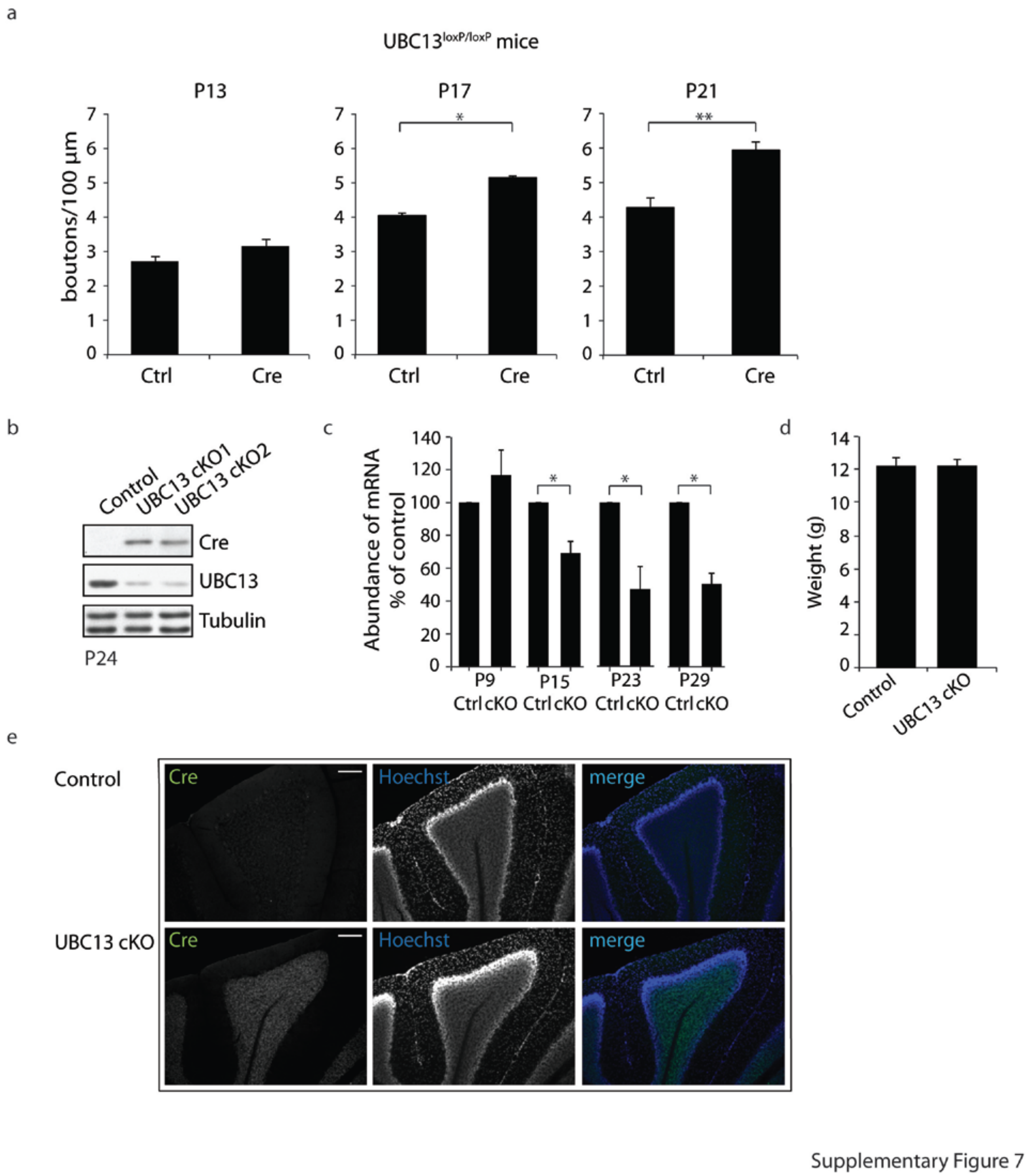

Supplementary Figure 7: Characterization of UBC13 conditional knockout mice

(a) P9 *UBC13*$^{loxP/loxP}$ mice were electroporated with the Cre expression plasmid or control vector together with the GFP expression plasmid and analyzed at different stages of synapse development. Quantification of the number of presynaptic boutons at different stages of development. Conditional knockout of UBC13 led to little or no difference in parallel fiber presynaptic boutons number at P13. By contrast, the number of parallel fiber presynaptic boutons was increased at P17 upon conditional knockout of UBC13 (*p<0.05, t test, n=3 mice), and this

difference was maintained at P21 (**$p<0.01$, t test, $n=3$ mice). (b) Lysates of the cerebellum from P24 mouse pups were immunoblotted with the UBC13, Cre and Tubulin antibodies. UBC13 protein was downregulated in UBC13 cKO mice compared to control mice. (c) Lysates of the cerebellum from P9, P15, P23 and P29 mice were subjected to qRT-PCR analyses using primers specific to UBC13, along with Gapdh. UBC13 mRNA was downregulated in the third postnatal week in UBC13 conditional knockout mice compared to control mice. (d) No difference in weight was found in P26-P28 UBC13 conditional knockout compared to control mice. (e) P30 cerebellar sections from UBC13 conditional knockout and control mice were subjected to immunohistochemistry using the Cre antibody and stained with the DNA dye hisbenzimide (Hoechst 33258). Conditional knockout of UBC13 in postmitotic granule neurons had little or no effect on the gross anatomy of the cerebellum (lobule 8 is shown). Scale bars=100μm.

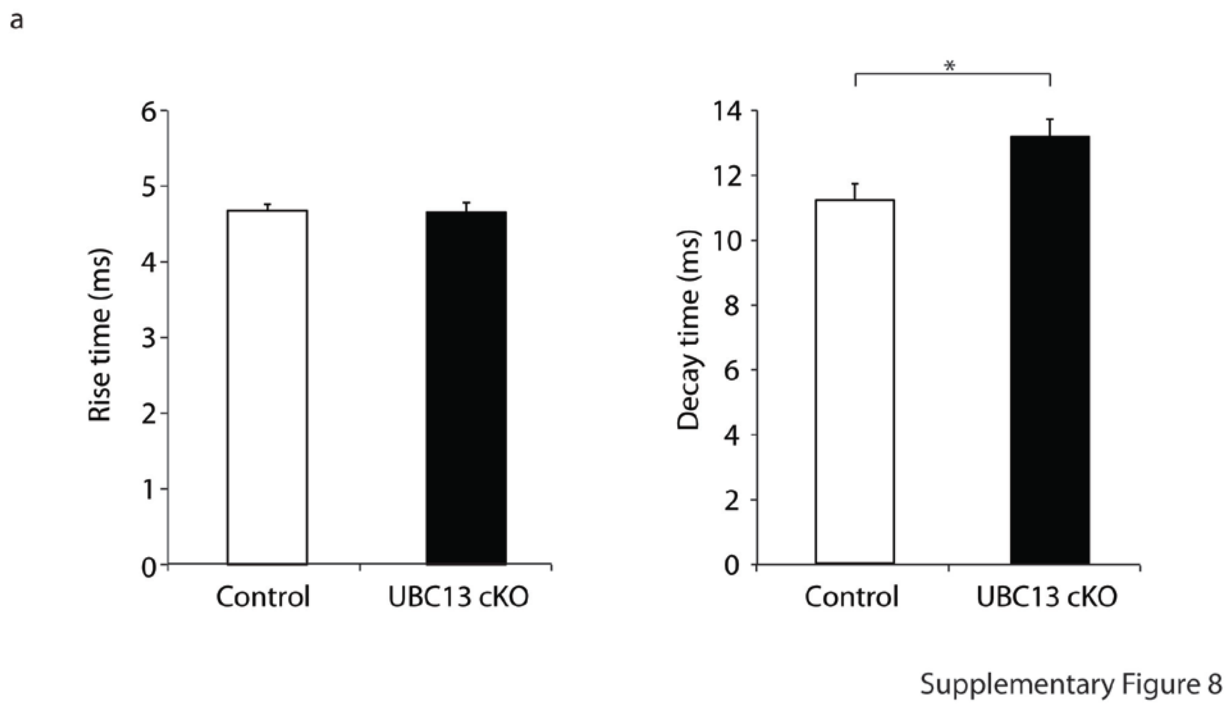

Supplementary Figure 8: Rise and decay time of mEPSCs in Purkinje neurons in UBC13 conditional knockout mice

(a) Little or no difference in the rise time was found in UBC13 conditional knockout and control mice. A modest increase in the decay time was found in UBC13 conditional knockout and control mice (*$p<0.05$, t test) (Supplementary Table 2).

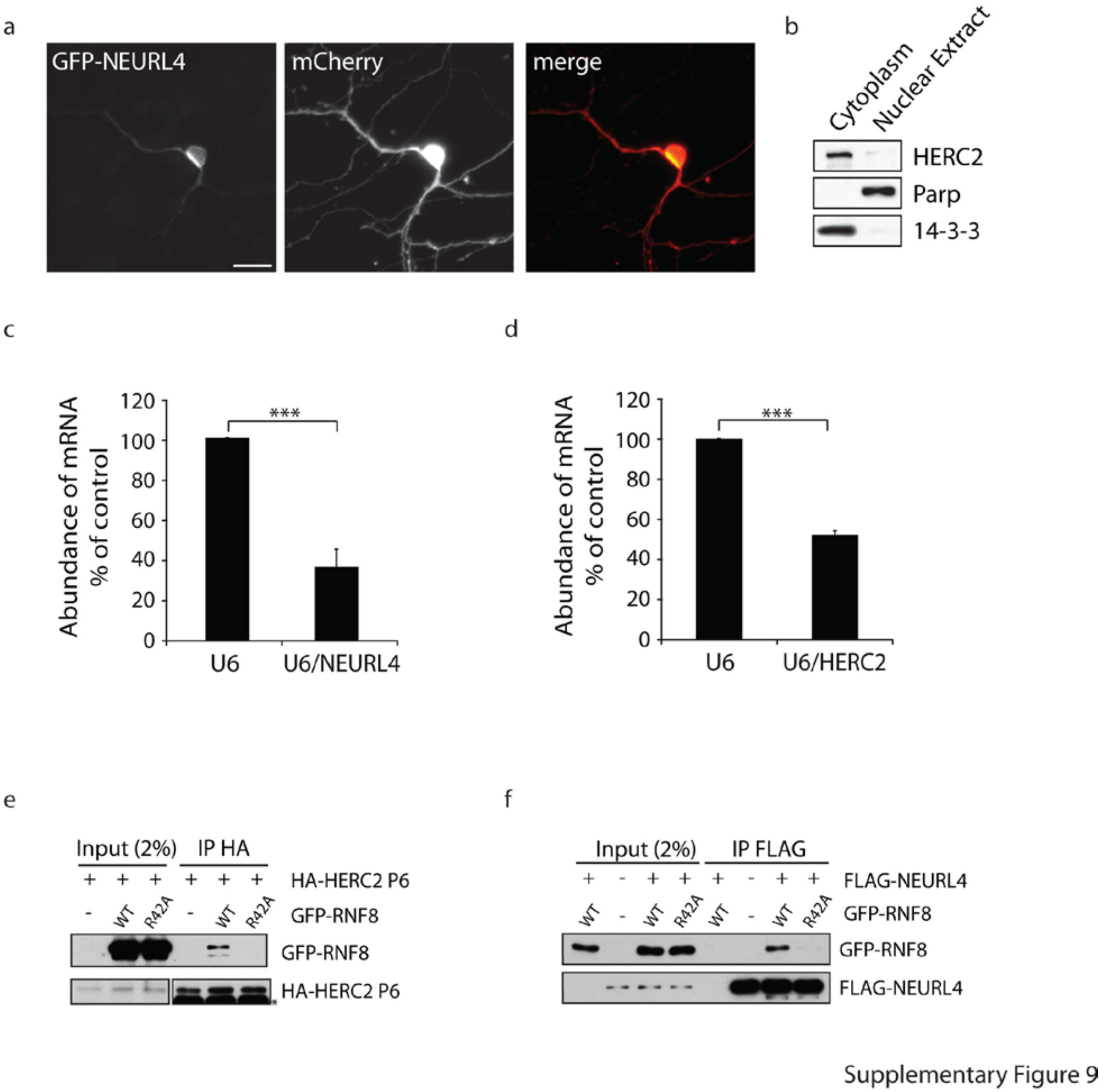

Supplementary Figure 9: Characterization of NEURL4 and HERC2 and interaction with RNF8

(a) The GFP-NEURL4 expression plasmid was electroporated together with an mCherry expression plasmid in primary granule neurons at DIV2. At DIV4, granule neurons were subjected to immunocytochemistry using the GFP and DsRed (mCherry) antibodies. GFP-NEURL4 was found localized in the cytoplasm in neurons. Scale bar=10μm. (b) Lysates of the cerebellum from P8 rat pups were fractionated and subjected to immunoblotting analyses using the HERC2, PARP, and 14-3-3 antibodies. Endogenous HERC2 was found predominantly in the cytoplasmic fraction. PARP and 14-3-3 were used to validate nuclear and cytoplasmic fractions, respectively. (c-d) Granule neurons were transfected with the U6/NEURL4 RNAi plasmid, U6/HERC2 RNAi plasmid or control U6 plasmid and subjected to qRT-PCR analyses using primers specific to NEURL4 or HERC2, along with Gapdh. The levels of each mRNA

normalized to Gapdh are shown relative to the control condition. NEURL4 RNAi and HERC2 RNAi induced the knockdown of endogenous NEURL4 (***p<0.005, t test, n=4 cultures) and endogenous HERC2 (***p<0.001, t test, n=3 cultures), respectively, in neurons. (e-f) Lysates of 293T cells transfected with an expression plasmid encoding HA-HERC2 P6, FLAG-NEURL4, GFP-RNF8 WT or GFP-RNF8 R42A. HA-HERC2 and FLAG-NEURL4 were immunoprecipitated with the HA or FLAG antibodies followed by immunoblotting with the GFP, HA or FLAG antibodies. Mutation of the FHA domain in RNF8 impaired the ability of RNF8 to form a complex with HERC2 and NEURL4 in neurons.

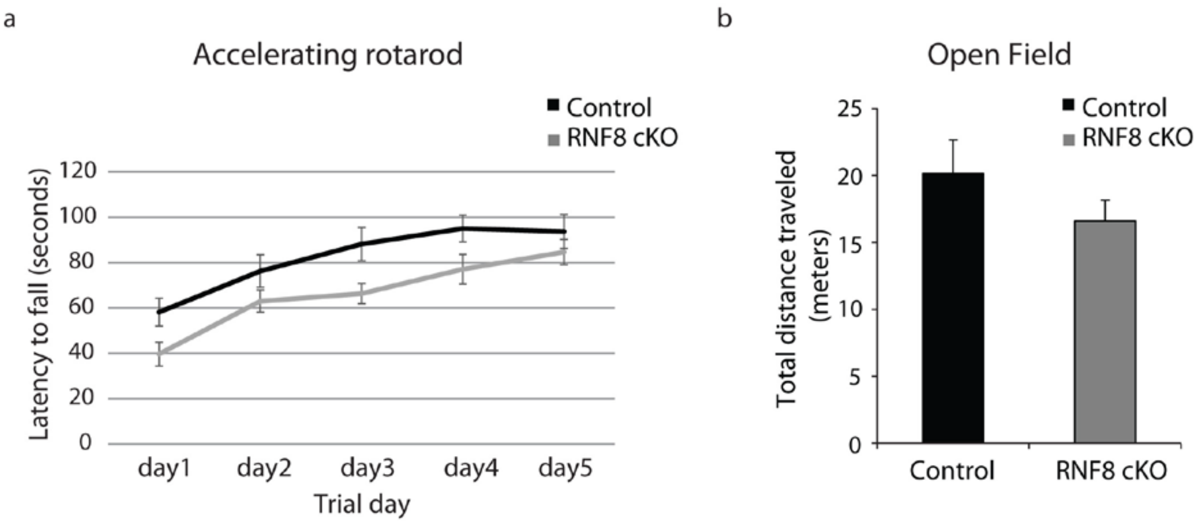

Supplementary Figure 10

Supplementary Figure 10: Accelerating rotarod and open field test
(a) A modest difference was observed in the ability of RNF8 conditional and control mice to learn in the accelerating rotarod test in which the latency to fall from accelerating rotarod increases over several days (control and RNF8 cKO, n=14 mice) (Supplementary Table 3). (b) Little or no difference was evident in the open-field test (control and RNF8 cKO, n=10 mice).

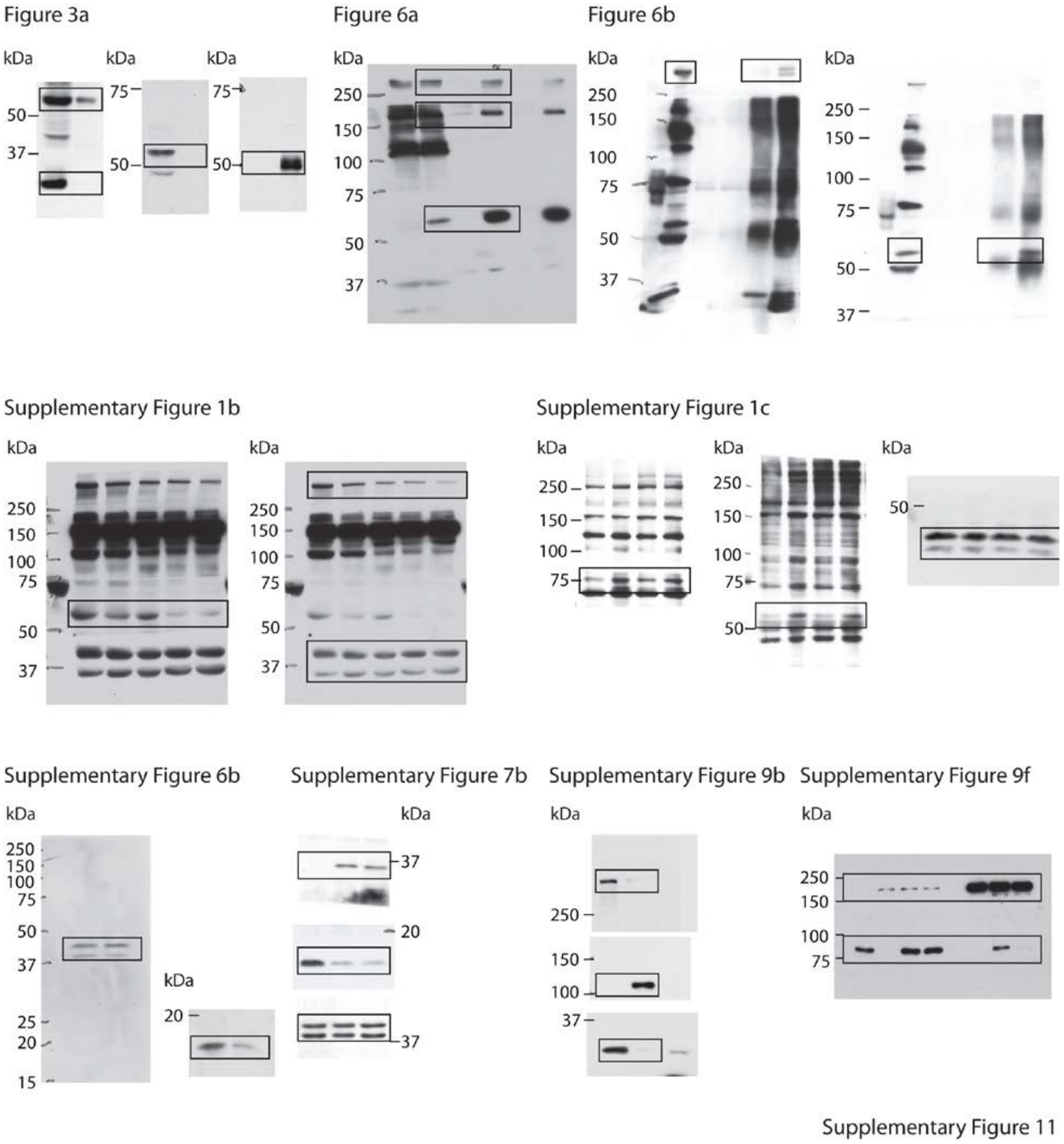

Supplementary Figure 11: The uncropped version of gel images

Supplementary Table 1: Rise and decay time of mEPSCs in RNF8 conditional knockout mice. Mean±s.e.m. and p-values.

	control	RNF8 cKO	p-value
**Rise (ms)**	4.26±0.12	4.57±0.15	0.12
**Decay (ms)**	9.36±0.60	10.93±0.59	0.068

Supplementary Table 2: Rise and decay time of mEPSCs in UBC13 conditional knockout mice. Mean±s.e.m. and p-values.

	control	UBC13 cKO	p-value
**Rise (ms)**	4.67±0.09	4.65±0.13	0.897
**Decay (ms)**	11.19±0.51	13.16±0.53	0.01

Supplementary Table 3: Rotarod performance. Mean±s.e.m. (in seconds) and p-values.

	control	RNF8 cKO	p-value
**day1**	39.64 ± 5.25	58.10 ± 6.15	0.031
**day2**	62.98 ± 4.85	76.24 ± 7.16	0.137
**day3**	66.36 ± 4.45	88.14 ± 7.34	0.017
**day4**	77.02 ± 6.52	95.05 ± 5.89	0.050
**day5**	84.64 ± 5.55	93.76 ± 7.48	0.337
